# Caspase-11 contributes to site-1 protease cleavage and SREBP1 activation in the inflammatory response of macrophages

**DOI:** 10.3389/fimmu.2023.1009973

**Published:** 2023-01-27

**Authors:** Yinglan Cheng, Ichiro Manabe, Sumio Hayakawa, Yusuke Endo, Yumiko Oishi

**Affiliations:** ^1^ Department of Biochemistry and Molecular Biology, Nippon Medical School, Bunkyo-ku, Japan; ^2^ Department of Systems Medicine, Graduate School of Medicine, Chiba University, Chiba, Japan; ^3^ Laboratory of Medical Omics Research, Kazusa DNA Research Institute, Kisarazu, Japan

**Keywords:** macrophage, SREBP (sterol regulatory element-binding protein) pathway, site-1 protease (S1P), caspase, inflammatory response

## Abstract

Sterol regulatory element-binding proteins (SREBPs) are key transcription factors that control fatty acid and cholesterol metabolism. As the major SREBP isoform in macrophages, SREBP1a is also required for inflammatory and phagocytotic functions. However, it is insufficiently understood how SREBP1a is activated by the innate immune response in macrophages. Here, we show that mouse caspase-11 is a novel inflammatory activator of SREBP1a in macrophages. Upon LPS treatment, caspase-11 was found to promote the processing of site-1 protease (S1P), an enzyme that mediates the cleavage and activation of SREBP1. We also determined that caspase-11 directly associates with S1P and cleaves it at a specific site. Furthermore, deletion of the *Casp4* gene, which encodes caspase-11, impaired the activation of S1P and SREBP1 as well as altered the expression of genes regulated by SREBP1 in macrophages. These results demonstrate that the caspase-11/S1P pathway activates SREBP1 in response to LPS, thus regulating subsequent macrophage activation.

## Introduction

Macrophages are essential players in the innate immune response due to their recognition of molecules associated with pathogens and cell damage *via* pattern recognition receptors (1). They not only participate in inflammatory processes but also crucially contribute to the resolution of inflammation and healing. Recent studies have shown that the regulation of cellular metabolism is tightly linked to the regulation of activation state in macrophages (2–4). Indeed, transcription factors that regulate metabolism, such as peroxisome proliferator-activated receptors (PPARs), liver X receptors (LXRs), and sterol regulatory element-binding proteins (SREBPs), have been shown to control inflammatory activation and resolution in macrophages (5–7), suggesting that they may act as key mediators of immunometabolic regulation in these cells.

SREBPs sense the intracellular lipid environment and control the expression of key genes in fatty acid and cholesterol metabolism to maintain lipid homeostasis. There are three different isoforms of SREBPs expressed in mammalian cells: SREBP1a and SREBP1c are encoded by the *Srebf1* gene and are generated by alternative splicing, and SREBP2 is encoded by *Srebf2*. Among these, SREBP1a is the major isoform in macrophages (8, 9). Our group and others have demonstrated the unique role of SREBP1a in connecting lipid metabolism and inflammatory responses. For instance, SREBP1a is activated around 4–6 h after TLR4 activation and upregulates fatty acid desaturation and elongation, which later contributes to the resolution of inflammation (10). SREBP1a was also shown to promote phagocytosis, at least in part, by controlling lipid synthesis (9). In addition, SREBP1a was reported to induce the expressions of *Nlrp1a*, which encodes a key component of the NLRP1 inflammasome, and *Cd5l*, the gene that encodes CD5L/AIM/Api6, a secreted glycoprotein that has been shown to have diverse roles in inflammatory processes (8, 11, 12). Thus, SREBP1 appears to be integral to both the active inflammatory and resolution functions of macrophages.

While SREBP1a plays important roles in the inflammatory responses of macrophages, it is insufficiently understood how it is activated under these conditions. SREBPs are synthesized as precursor proteins in the endoplasmic reticulum (ER). In hepatocytes, the level of cellular cholesterol determines SREBP activity. When cholesterol levels are high, SREBPs are retained in the ER membrane as a complex with two membrane proteins, SREBP cleavage activating protein (SCAP) and insulin-induced gene (INSIG). Upon cholesterol deprivation, however, the SCAP-SREBP complex is released from INSIG and is translocated to the Golgi apparatus by coat protein II (COPII)-coated vesicles. In the Golgi, the mature form of SREBP is generated by sequential proteolytic cleavage mediated by site-1 protease (S1P/MBTPS1) and site-2 protease (S2P/MBTPS2) and is released from the Golgi membrane. The mature SREBP then translocates into the nucleus, where it transactivates its downstream target genes (13). However, in macrophages, cellular free cholesterol levels are increased with inflammatory activation (14), so it remains unclear how SREBP1a is activated under these conditions.

Cysteine-dependent aspartate-specific proteases (caspases) are a family of proteases that play pivotal roles in apoptosis and inflammation, and they can be broadly classified based on their association with these processes (apoptosis: caspases-3, -6, -7, -8, -9, and -10; inflammation: caspases-1, -4, -5, -11, and -12) (15). Inflammatory caspases drive inflammatory cytokine secretion and pyroptosis. In mice, the inflammatory caspases are caspases-1, -11, and -12. Mouse caspase-11, which is encoded by *Casp4*, has a high degree of sequence similarity to human caspases-4 and -5 (CASP4 and CASP5), yet it remains unclear which of these proteins is its functional human ortholog. Caspase-11 is activated by mechanisms that are different from those activating caspase-1, which require the formation of an inflammasome complex (16). Indeed, caspase-11, which is found as a pro-enzyme under steady state, directly binds to internalized LPS and undergoes oligomerization and proximity-induced autoproteolysis that cleaves the pro-domain from the catalytic domain (17–19), resulting in enzyme activation.

There are several reports showing that caspases are involved in the activation of SREBPs. Caspase-1 activates SREBP2 in response to toxin-induced membrane damage through the assembly of the NLRP3 inflammasome in fibroblasts (20). In this setting, caspase-1 activates SREBPs in a manner that depends on S1P and S2P, though the precise role of caspase-1 remains unknown. Moreover, in ethanol-exposed hepatocytes, TNF-α activates caspases-4 and -12, which then cleave SREBP1 at locations other than the S2P-mediated cleavage site to mediate its activation (21).

Based on these previous observations, we hypothesized that inflammatory caspases might have a role in mediating SREBP1a activation during the inflammatory response in macrophages. In agreement with previous findings (22), we found that among the inflammatory caspases, only caspase-11 was activated by LPS in macrophages. Using a series of *in vitro* experiments, we have shown that caspase-11 directly recognizes and activates S1P, thereby triggering the activation of SREBP1a in response to LPS in macrophages. Furthermore, our data suggest that caspase-11-mediated SREBP1 activation is necessary for generating a proper inflammatory response.

## Results

### Caspase-11 is required for SREBP1 activation in the inflammatory response to LPS

**Figure 1:**
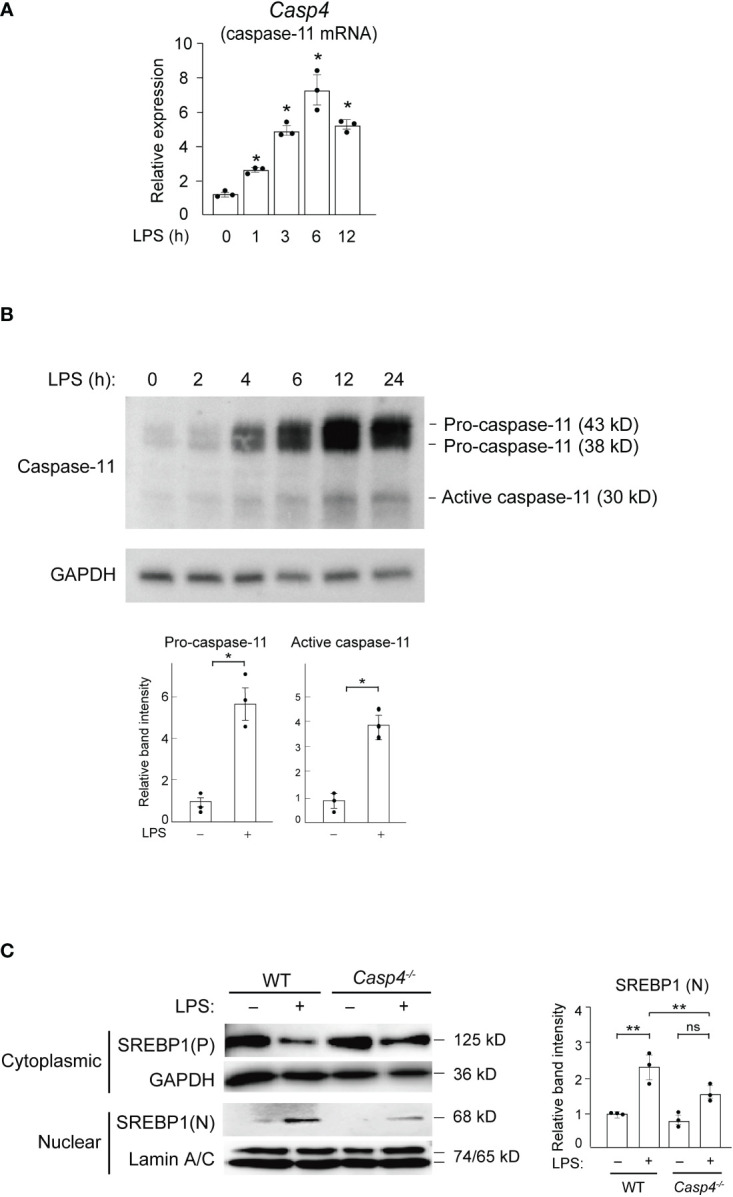
Caspase-11 is required for nuclear translocation of SREBP1 in response to LPS. **(A, B)** BMDMs were treated with 100 ng/ml LPS for the indicated times and subjected to quantitative RT-PCR **(A)** or western blot **(B)** analysis. In A, *Casp4* mRNA expression levels were first normalized to those of *Gapdh* and then to the level *Casp4* expression at 0 h of LPS. Data represent means ± SEM. Significance was determined using one-way ANOVA followed by Tukey’s *post hoc* test versus 0 (h) *P < 0.05. *n*=3 for each time point. In B, whole cell lysates of LPS-treated BMDMs were subjected to western blotting for caspase-11 and GAPDH. The relative band intensities corresponding to pro-caspase-11 and active caspase-11 in untreated BMDMs and those treated with LPS for 6 h are shown in the bar graphs. *n*=3. Significance was determined using Student’s *t*-tests. *P < 0.05. **(C)** BMDMs from WT and *Casp4^–/–^* mice were treated with LPS for 6 h, and cytoplasmic lysates and nuclear extracts were subjected to western blotting for SREBP1. A representative western blot of three independent experiments is shown. P, precursor; N, nuclear. The relative band intensities corresponding to nuclear SREBP1 are shown in the bar graph. *n*=3. Significance was determined using one-way ANOVA followed by Tukey’s test for multiple comparisons. **P < 0.01; ns: not significant.

To test the hypothesis that inflammatory caspases might mediate LPS-induced activation of SREBP1a, we first focused on caspase-11 because, unlike caspase-1, which is not activated by LPS stimulation alone (23), caspase-11 can be activated by binding to internalized LPS (17, 24–27). To examine caspase-11 expression in response to LPS, we stimulated bone marrow-derived macrophages (BMDMs) with 100 ng/ml of LPS and found that the mRNA expression of *Casp4*, which encodes caspase-11, started to increase at 1 h and reached its peak at 6 h after treatment (**Figure 1A**). Furthermore, in line with a previous report (28), we found increased levels of both the precursor (43 and 38 kD) and active (30 kD) forms of caspase-11 following treatment with LPS (**Figure 1B**), suggesting that LPS can activate caspase-11 in macrophages. In contrast, while the mRNA and precursor protein expressions of other inflammatory caspases (caspases-1 and -12) were also increased in BMDMs following LPS treatment, their active forms were not detected in western blot experiments (**Supplemental Figures 1A, B**). These results demonstrate that only caspase-11, but not caspase-1 or -12, is activated by LPS in macrophages (22, 29 30). Hereafter, we will use caspase-11 to refer to the protein and *Casp4* to refer to the mRNA and gene because it remains unclear whether mouse caspase-11 is the ortholog of human CASP4 or CASP5.

To determine whether caspase-11 is involved in the SREBP1 pathway, we performed experiments using BMDMs from wild-type (WT) and *Casp4* knockout mice (31, 32). In untreated, WT BMDMs, we found that the majority of the SREBP1 existed in the cytosolic precursor form and that nuclear SREBP1 was barely detectable (**Figure 1C**). However, following 6 h of LPS stimulation, we observed an increased amount of the nuclear form of SREBP1, with a concomitant decrease in the amount of cytosolic SREBP1 precursor protein, indicating that LPS activated SREBP1. Compared to WT cells, the level of nuclear SREBP1 detected after 6 h of LPS was lower in *Casp4* ^-/-^ BMDMs (**Figure 1C**), demonstrating that caspase-11 is important for the LPS-induced nuclear translocation of SREBP1 in BMDMs.

Previous studies have shown that, based on mRNA levels, SREBP1a is the major isoform of SREBP1 in macrophages (8). However, the available anti-SREBP1 antibodies do not distinguish between the SREBP1 isoforms. Consequently, we could not conclude that SREBP1a specifically translocated to the nucleus in response to LPS in our experiments. However, the abundance of *Srebf1a* mRNA strongly suggests that the major nuclear isoform was SREBP1a.

We found that *Casp4* deletion did not affect the levels of *Srebf1a* mRNA (**Supplemental Figure 2**) or cytoplasmic SREBP1 in untreated BMDMs (**Figure 1C**). Moreover, LPS increased *Srefb1a* expression in WT BMDMs, as previously reported (33), while *Casp4* deletion tended to decrease the upregulation of *Srebf1a* expression induced by LPS, though the difference did not reach statistical significance (*P* = 0.0625) (**Supplemental Figure 2**). This attenuation of *Srebf1a* induction in *Casp4* ^-/-^ cells might be due to reduced SREBP1 activity because the *Srebf1* promoter is autoregulated by SREBP (34, 35).

### Caspase-11 is required for S1P activation

**Figure 2:**
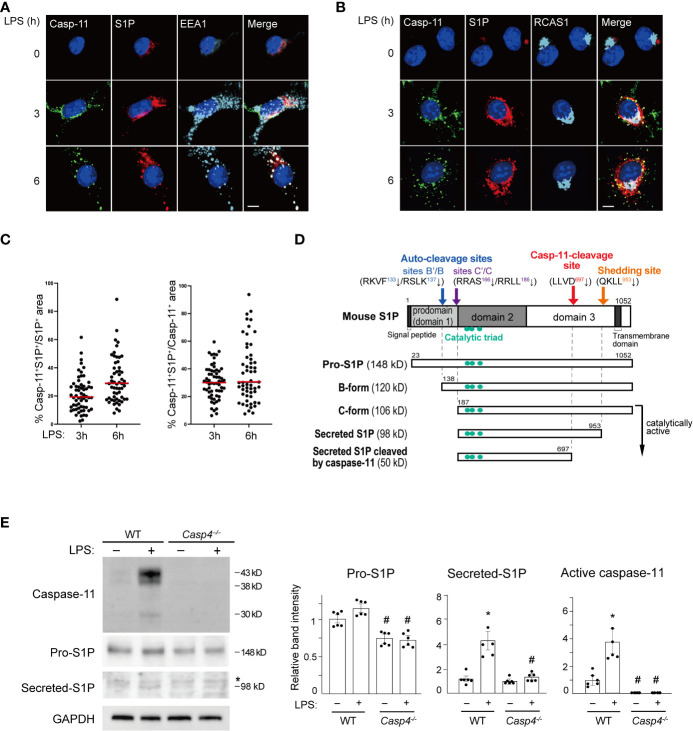
Caspase-11 promotes S1P processing. **(A, B)** BMDMs were treated with LPS for the indicated times. Caspase-11 (green), S1P (red), and the endosome marker EEA1 in A or the Golgi apparatus marker RCAS1 in B (light blue) were visualized by immunofluorescence staining. Nuclei were counterstained with DAPI. Scale bar, 10 mm. **(C)** Fractions of caspase-11- and S1P- double positive areas within S1P- or Caspase-11-positive areas. Sixty cells per each condition were analyzed. Caspase-11 signal was not detected without LPS stimulation. **(D)** A schematic representation of the processing of mouse S1P. **(E)** BMDMs from WT and *Casp4^–/–^* mice were treated with LPS for 6 h. Caspase-11, S1P, and GAPDH expression in whole cell lysates were detected by western blotting. Asterisks indicate non-specific bands. The relative band intensities corresponding to pro-S1P, secreted S1P, and active caspase-11 are shown in the bar graphs. *n*=6. Significance was determined using one-way ANOVA followed by Tukey’s test for multiple comparisons. *P < 0.05 vs. untreated of the same genotype, ^#^ *P* < 0.05 vs. WT of the same treatment.

We next investigated the mechanism behind how caspase-11 activates SREBP1 by looking at the intracellular distribution of caspase-11 using immunofluorescence staining. Caspase-11 protein was barely visible in untreated BMDMs. However, after LPS treatment, caspase-11 signal was increased in the cytoplasm with a punctate staining pattern and was partly localized to EEA1^+^ endosomes and RCAS^+^ Golgi apparatus (**Figures 2A, B**). In contrast, the caspase-11 staining patterns observed did not appear to correlate with the staining patterns of known ER (PDI) or lysosome (LAMP1) markers (**Supplemental Figures 3A** and **3B**).

Because caspase-11 is partly localized to the Golgi but not the ER, it is likely involved in the SREBP1 activation process that takes place in the Golgi. S1P, which cleaves SREBP1, is also processed to its active form in the Golgi (36). Consequently, we decided to investigate a possible interaction between caspase-11 and S1P. As previously reported (37, 38), we found that S1P was mainly localized to the endosome and Golgi apparatus in BMDMs (**Figures 2A, B**). Furthermore, our immunofluorescence staining showed that both the caspase-11 and S1P signals were increased in BMDMs after LPS stimulation. Their signals were also partly colocalized in the endosome and Golgi after 3 and 6 h of treatment with LPS (**Figures 2A–C**).

After determining that caspase-11 and S1P were colocalized in BMDMs after LPS treatment, we next addressed whether caspase-11 is important for the activation of S1P under these conditions. S1P is generated as an inactive precursor (pro-S1P) and a series of processing steps produces active enzyme (**Figure 2D**) (36, 39, 40). We found that the mRNA level of *Mbtps1*, the gene that encodes S1P, was increased by LPS treatment in BMDMs and was not affected by the lack of *Casp4* (**Supplemental Figure 4**). In contrast, the level of pro-S1P protein (148 kD) was lower at baseline in *Casp4* ^-/-^ BMDMs than in WT BMDMs. Upon LPS treatment, we repeatedly found that pro-S1P was modestly increased in WT BMDMs, though the difference, as determined by densitometry analysis, did not reach statistical significance (**Figure 2E**). We also found that the expression of the secreted form of S1P (98 kD) was increased more than the expression of pro-S1P in WT cells. Accordingly, while the increase in pro-S1P was modest, upregulation of Mbtps1 expression is likely to have contributed to the increase in the active form of S1P. In contrast, we did not observe significant increases in the expressions of either the pro- or secreted forms of S1P in *Casp4* ^-/-^ cells treated with LPS (**Figure 2E**). These results suggest that caspase-11 is required for the processing and activation of S1P.

In addition to S1P, SREBP cleavage activating protein (SCAP) and site-2 protease (S2P/MBTPS2) are also involved in the translocation and activation of SREBP1. Therefore, we assessed whether these proteins may also affect the activation of caspase-11. We found that caspase-11, including the active form, was induced by LPS in BMDMs regardless of *Scap* or *Mbtps2* (S2P) knockdown (**Supplemental Figure 5A, B**), suggesting that SCAP and S2P are dispensable for the activation of the caspase-11 pathway. In addition, deletion of *Casp4* did not affect *Scap* or *Mbtps2* expression (**Supplemental Figure 5C**).

Previous studies have reported that the PI3K/AKT/mTORC1/S6K pathway promotes SREBP1 processing and activation, though the precise molecular mechanism behind how this pathway affects SREBP1 processing remains unclear (41). To test for possible crosstalk between the PI3K/AKT/mTORC1/S6K and caspase-11 pathways, we first analyzed the effects of *Casp4* deletion on S6K and S6 activation. As previously reported (9), we found that LPS promoted the phosphorylation of S6K and S6, and that this activation was inhibited by LY204002 (PI3K inhibitor) and rapamycin (mTORC1 inhibitor) (**Supplemental Figures 6A, B**). We also observed that in *Casp4* ^-/-^ BMDMs, S6K and S6 phosphorylation were not suppressed following LPS stimulation, suggesting that caspase-11 is dispensable for the LPS-induced activation of the PI3K/AKT/mTORC1/S6K pathway (**Supplemental Figures 6A, B**). Next, we analyzed the effects of PI3K and mTORC1 inhibitors on caspase-11 proteins and found that the expression levels of both the pro- and active forms were increased by LPS in the presence of LY294002 and rapamycin (**Supplemental Figures 6C, D**). Collectively, these findings strongly suggest that the caspase-11/S1P pathway can be activated independently of the PI3K/AKT/mTORC1/SRK pathway in response to LPS.

### Caspase-11 physically associates with S1P

**Figure 3:**
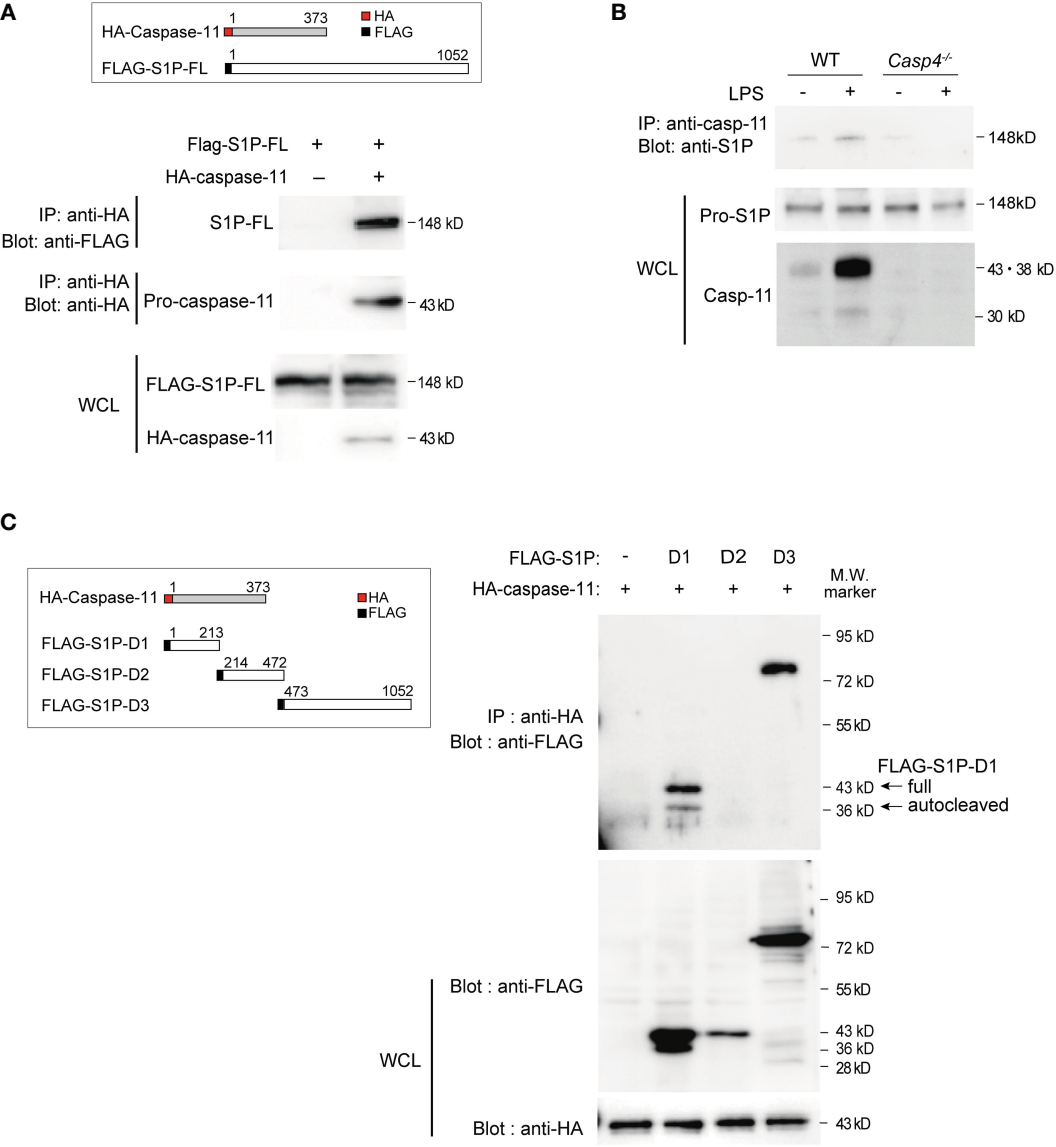
Caspase-11 interacts with S1P domains 1 and 3. **(A)** HEK293T cells were transfected with plasmids expressing HA-tagged mouse caspase-11 and full-length FLAG-tagged mouse S1P. Twenty-four hours after transfection, whole cell lysates (WCLs) were immunoprecipitated with HA antibody and immunoblotted with FLAG or HA antibody to detect the interaction between S1P and caspase-11. The protein expression levels of FLAG-tagged S1P and HA-tagged caspase-11 within the WCLs were detected by western blotting using anti-FLAG or anti-HA antibodies, respectively. **(B)** Endogenous S1P and caspase-11 protein directly interact. BMDMs were stimulated for 6 h with or without LPS before preparing whole cell lysates. The S1P protein pulled down by the caspase-11 antibody was detected by western blotting using an anti-S1P antibody. **(C)** HEK293T cells were transfected with HA-tagged mouse caspase-11 and deletion mutants of FLAG-tagged mouse S1P. FLAG empty vector was used as a control. Twenty-four hours after transfection, cells were harvested and WCLs were subjected to immunoprecipitation with anti-HA antibody and subsequent western blot analysis with anti-FLAG antibody. The protein expression levels of each FLAG-tagged S1P mutant and HA-tagged caspase-11 in the WCLs were shown by western blotting (bottom two blots). In cells transfected with plasmids expressing FLAG-S1P-D1 and HA-caspase-11, two bands were detected by the anti-FLAG antibody, corresponding to the full D1 fragment (43 kD) and an autocatalyzed form (36 kD).

To examine how caspase-11 is involved in S1P activation, we first tested whether caspase-11 directly interacts with S1P using coimmunoprecipitation experiments. For these assays, FLAG-tagged S1P- and HA-tagged caspase-11-expressing vectors were transfected into HEK293T cells. Whole-cell lysates were then prepared, proteins were immunoprecipitated with an antibody against the HA tag, and association with S1P was detected by western blot using an anti-FLAG antibody (**Figure 3A**). Our results indicated that caspase-11 directly associates with S1P. Moreover, additional coimmunoprecipitation experiments showed that endogenous pro-S1P physically associated with caspase-11 in LPS-treated BMDMs (**Figure 3B**).

To further characterize the domains that mediate the interaction between caspase-11 and S1P, we generated plasmids expressing FLAG-tagged versions of the three different domains of mouse S1P (domains 1, 2, and 3; **Figures 2D**, **3C**). Domain 1 consists of the pro-domain (36, 39), domain 2 is the catalytic domain containing the catalytic triad (Asp, His, Ser), and domain 3 consists of the ectodomain, as well as transmembrane and internal domains (39). These plasmids were then co-transfected with HA-caspase-11 in HEK293T cells, and lysates were subjected to immunoprecipitation with anti-HA antibody followed by western blotting with anti-FLAG antibody to detect protein interactions. We found that while S1P-domains 1 and 3 coimmunoprecipitated with caspase-11, domain 2 did not (**Figure 3C**). Collectively, our results revealed that caspase-11 directly binds to S1P *via* two regions: domain 1 and domain 3.

### Caspase-11 promotes the processing of S1P into its active form

Thus far, our data has shown that deletion of *Casp4* reduces the generation of the active, secreted form of S1P in BMDMs (**Figure 2E**) and that caspase-11 physically interacts with S1P (**Figures 3A, B**), suggesting that caspase-11 is important for the processing of S1P. S1P is synthesized as an inactive transmembrane precursor protein and requires multiple proteolytic events to become catalytically active (**Figure 2D**). S1P protein is first cleaved by a signal peptidase to generate pro-S1P (amino acids 23-1052) (36, 39, 40). Generation of pro-S1P exposes the N-terminal pro-domain, which undergoes sequential autocatalytic processing in the ER (39, 42) at the RKVF^133^↓/RSLK^137^↓ and RRAS^166^↓/RRLL^186^↓ cleavage sites to generate the B-form (120 kD) and C-form (106 kD), respectively (36, 37). The membrane-bound C-form is further cleaved at the shedding site (KHQKLL^953^↓) to become the secreted form of S1P (98 kD) in Golgi (36, 39, 43, 44) (**Figure 2D**). The secreted form of S1P cleaves the luminal loop of SREBP precursors (45) and can also be secreted into the extracellular space (46).

**Figure 4:**
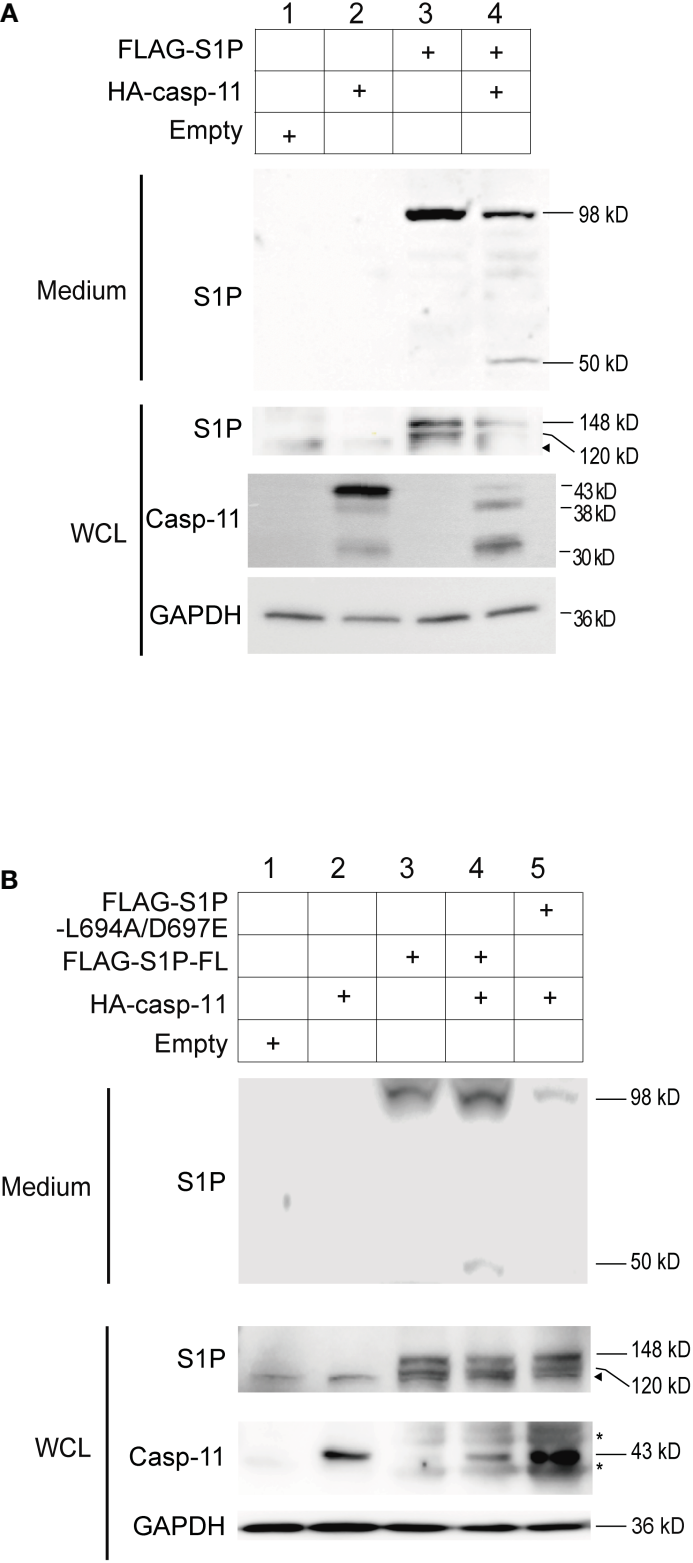
Caspase-11 is necessary for the formation of the secreted form of S1P. **(A)** HEK293T cells were transfected with plasmids containing FLAG-tagged full-length S1P and HA-tagged caspase-11. Twenty-four hours after transfection, WCLs and culture medium were collected, and the levels of the processed forms of S1P were assessed by western blotting using an anti-S1P antibody. **(B)** HEK293T cells were transfected with plasmids containing WT FLAG-S1P or a FLAG-S1P carrying mutations in the caspase-11-binding sequence (L694A/D697E). Twenty-four hours after transfection, culture medium and WCLs were collected and subjected to western blotting using anti-S1P and anti-caspase-11 antibodies. The arrowhead indicates the band corresponding to endogenous S1P. Asterisks indicate non-specific bands.

To test whether caspase-11 promotes the catalytic cleavage of precursor-S1P into its active form, HEK293T cells were transfected with plasmids expressing full length S1P and caspase-11. Western blot analysis of whole-cell lysates from cells expressing S1P alone showed the presence of two bands corresponding to pro-S1P (148 kD A-form) and the 120 kD B-form (36, 39, 40) of S1P (**Figure 4A**, lane 3). The C-form of S1P was not detected, presumably because of immediate autocatalysis at the shedding site. Secreted S1P (98 kD) was also detected in the culture media, indicating that HEK293T cells possess the intrinsic machinery required for the processing of pro-S1P to its 98 kD secreted form(**Figure 4A**, lane 3), which is in line with a previous report (42). When S1P and caspase-11 were coexpressed in HEK293T cells, the amounts of pro-, B-form, and secreted S1P were decreased, and an additional 50 kD fragment of S1P was detected in the medium (**Figure 4A**, lane 4). These results demonstrate that caspase-11 promotes the processing of S1P.

Based on our results, we next sought to identify the specific site in mouse S1P that is cleaved by caspase-11. Caspases generally recognize the tetrapeptidic sequence L(E/L/M)VD and have a stringent specificity for cleaving the peptide bond that is C-terminal to the aspartic acid (D) residue. Through our analysis of the mouse S1P protein sequence, we identified a site, ^694^LLVD^697^, within domain 3 that fits the caspase recognition motif and is conserved in mouse, human, rat, and hamster (**Figure 2D**, **Supplemental Figure 7**). Cleavage at this site is predicted to generate a 50 kD fragment containing the catalytic domain.

To test whether S1P is cleaved at our predicted caspase-11 recognition site, we generated an expression plasmid encoding a FLAG-tagged mouse S1P L694A/D697E mutant in which the leucine (L at position 694) and aspartic acid (D at position 697) residues were substituted with alanine (A) and glutamic acid (E), respectively, and expressed it in HEK293T cells along with full length caspase-11. While the 50 kD fragment was detected in the medium of cells expressing WT S1P and caspase-11, it was not observed in the medium of cells expressing the L694A/D697E mutant and caspase-11 (**Figure 4B**, lanes 4 and 5). These results strongly suggest that caspase-11 cleaves S1P at ^694^LLVD^697^. In addition, while the secreted 98 kD form of S1P was detected in the medium of cells expressing either the WT or the L694A/D697E S1P mutant (**Figure 4B**, lanes 3-5), we repeatedly found that the secreted form was less abundant in the medium of cells expressing the mutant. These findings suggest that the interaction of caspase-11 with S1P at the ^694^LLVD^697^ site may be important for promoting the processing of S1P.

### Caspase-11 is important for SREBP1-dependent gene regulation

**Figure 5:**
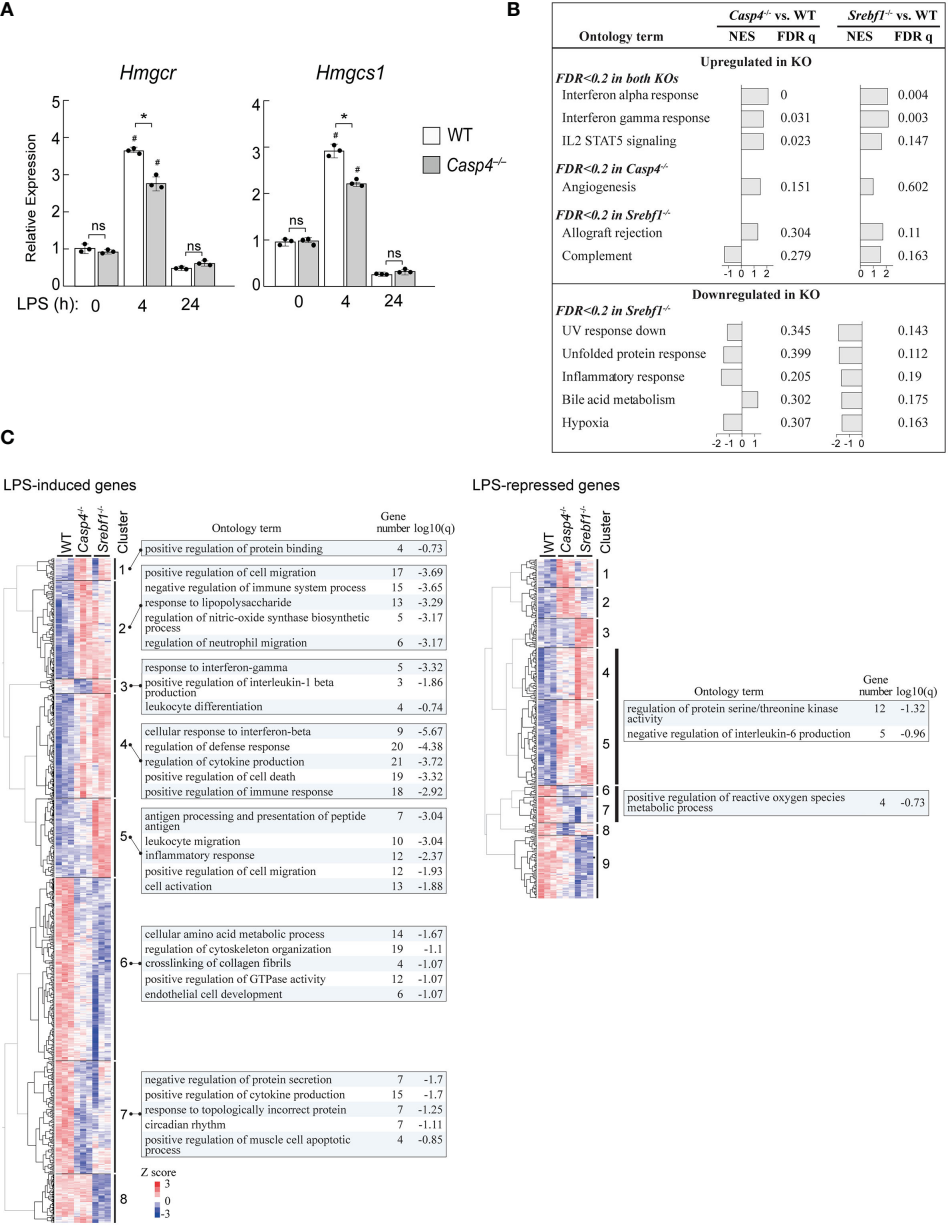
*Casp4* and *Srebf1* deletions affect LPS-dependent gene expression. **(A)** BMDMs obtained from WT or *Casp4* ^-/-^ mice were stimulated with LPS for the indicated times. The mRNA expression levels of *Hmgcr* and *Hmgcs1* were quantified by qPCR. mRNA levels were first normalized to those of *Gapdh* and then to the expression levels of the target genes in untreated, WT BMDMs. The bars represent means ± SEM. *n*=3 for each time point. Significance was determined by one-way ANOVA followed by Tukey’s multiple comparison test. *P < 0.05, ^#^ *P* < 0.05 compared to the 0 h value for the same genotype. ns, not significant. **(B, C)** WT, *Casp4* ^-/-^, and *Srebf1* ^-/-^ BMDMs were treated with LPS for 4 h and their transcriptomes were analyzed by RNA-seq. *n*=3 in each group. In B, enrichment analysis was performed using GSEA (47). MSigDB hallmark gene sets enriched in *Casp4* ^-/-^ and/or *Srebf1* ^-/-^ BMDMs compared to WT BMDMs (FDR < 0.2) are shown. NES, normalized enrichment score. Note that no gene sets reached FDR < 0.2 for *Casp4* ^-/-^ BMDMs. In C, the expression levels of genes significantly (FDR < 0.05) upregulated or downregulated in WT BMDMs and differentially expressed between WT and either *Casp4* ^-/-^ or *Srebf1* ^-/-^ BMDMs treated with LPS for 4 h (FDR < 0.05) were hierarchically clustered. Heatmaps of the standardized expression levels of 870 LPS-induced and 294 LPS-repressed genes in each cell after 4 h of LPS are shown. The top 5 gene ontology terms or reactome gene sets enriched (FDR < 0.2) for each cluster are also shown. Gene number is the number of genes in the cluster with membership in the ontology term. Note that gene sets were not identified for each cluster of LPS-repressed genes. The sets of genes enriched in clusters 4 and 5 as well as 6 and 7 of the LPS-repressed genes are shown. Genes belonging to each cluster are shown in **Supplemental Table 2**.

Our data suggests that caspase-11 is required for the activation of SREBP1 during the innate immune response of macrophages to LPS. We therefore investigated the ability of caspase-11 to alter gene expression programs during the LPS-mediated activation of BMDMs. Our previous ChIP-seq (GSE79423) experiment in mouse primary macrophages demonstrated that SREBP1 binds to the promoters of the *Hmgcr* and *Hmgcs1* genes, which encode enzymes in the cholesterol biosynthesis pathway (**Supplemental Figure 8**). In addition, while H3K27Ac deposition, which reflects enhancer activation state, in the regions surrounding the SREBP1 binding sites in the *Hmgcr* and *Hmgcs1* genes in WT BMDMs was increased after treatment with the TLR4-specific ligand Kdo2-lipid A (KLA), this increase was partially inhibited in *Srebf1* ^-/-^ BMDMs (**Supplemental Figure 8A, B**). These observations clearly demonstrate that *Hmgcr* and *Hmgcs1* are direct target genes of SREBP1 in macrophages. Therefore, we analyzed the effects of *Casp4* deficiency on *Hmgcr* and *Hmgcs1* expression (**Figure 5A**). After 4 h of LPS stimulation, the mRNA expressions of *Hmgcr* and *Hmgcs1* were upregulated in WT BMDMs; however, there was less induction of these genes in *Casp4^-/-^* BMDMs, demonstrating that caspase-11 is involved in activating the expressions of these genes in response to LPS.

To gain further insights into the role of caspase-11 in SREBP1-dependent gene regulation, we compared the effects of deletions of *Casp4* and *Srebf1* on the transcriptomes of BMDMs treated with LPS for 4 h using RNA-sequencing (RNA-seq) assays. Gene set enrichment analysis (GSEA) (47) of the RNA-seq data revealed that deletion of *Casp4* and *Srebf1* both affected the expressions of sets of genes related to inflammation, such as genes involved in the interferon response and IL2 STAT5 signaling, in LPS-treated BMDMs (**Figure 5B**). Furthermore, *Srebf1* deletion resulted in the downregulation of genes related to cellular stress responses, such as the UV response, the unfolded protein response, and hypoxia, which also tended to be downregulated in *Casp4* ^-/-^ BMDMs (**Figure 5B**).

To further examine the relationship between the changes in gene expression observed in *Casp4* ^-/-^ and *Srebf1* ^-/-^ BMDMs, we compared the expression levels of LPS-induced and -repressed genes that were differentially expressed in either *Casp4* ^-/-^ or *Srebf1* ^-/-^ BMDMs, as compared to WT cells. Heatmaps of hierarchically clustered gene expression data indicated extensive similarities between the effects of *Casp4* and *Srebf1* deletion on the expression of LPS-responsive genes, particularly for the LPS-induced genes (**Figure 5C**), supporting the notion that caspase-11 is important for the activation of SREBP1 after LPS stimulation. Enrichment analysis using Metascape (48) showed that the LPS-induced genes that were commonly upregulated in both *Casp4* ^-/-^ and *Srebf1* ^-/-^ BMDMs (clusters 2 and 4) were primarily related to inflammation, cell migration, and cell death. In contrast, the LPS-induced genes that were downregulated in the knockout cells (clusters 6 and 7) were mostly related to metabolism, cytokine production, and cytoskeleton organization (**Figure 5C**, left). Analysis of the LPS-repressed genes that were upregulated in the knockout cells (clusters 4 and 5) resulted in enrichment of the ontology terms “regulation of protein serine/threonine kinase activity” and “negative regulation of interleukin-6 production,” while those genes that were downregulated in the knockout cells (clusters 6 and 7) were enriched for the “positive regulation of reactive oxygen species metabolic process” ontology term (**Figure 5C**, right). Taken together, these results suggest that *Casp4* has a strong impact on SREBP1-dependent gene regulation in LPS-activated macrophages.

### Human CASP5 mediates the LPS response in human THP-1 macrophages

**Figure 6:**
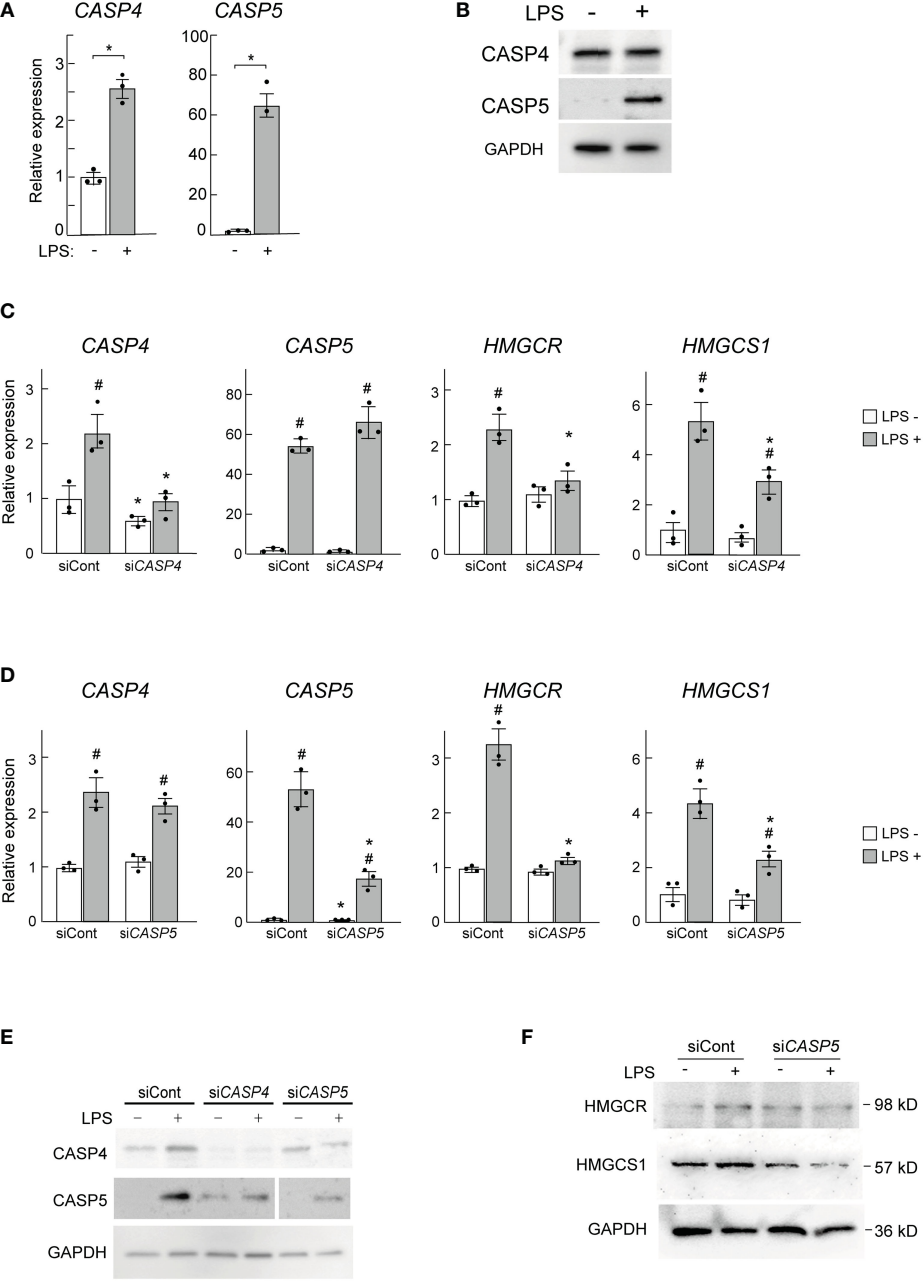
*CASP5* deletion affects LPS-dependent gene expression in THP-1 cells. **(A, B)** THP-1 cells were stimulated with LPS (500 ng/ml) for 0 and 6h *CASP4* and *CASP5* mRNA expression levels were analyzed by qPCR **(A)**, and CASP4 and CASP5 protein expression levels were analyzed by western blotting **(B)**. **(C, D)** THP-1 macrophages were transfected with control siRNA (siCont) or siRNAs against *CASP4* **(C)** or *CASP5* **(D)**. The expression levels of *CASP4*, *CASP5, HMGCR*, and *HMGCS1* mRNAs in *CASP4* **(C)** and *CASP5* **(D)** knockdown cells were analyzed by qPCR. Significance was determined by one-way ANOVA followed by Tukey’s multiple comparison test. *P < 0.05 vs. the siCont-transfected cells without LPS, ^#^ *P* < 0.05 vs. the cells transfected with the same siRNA without LPS. **(E)** CASP4 and CASP5 protein levels in *CASP4* and *CASP5* knockdown cells were analyzed by western blotting. **(F)** HMGCR and HMGCS1 protein levels in *CASP5* knockdown cells were analyzed by western blotting.

Finally, we asked whether the human ortholog of mouse caspase-11 might also be required for the induction of *HMGCR* and *HMGCS1* in human macrophages. Because mouse caspase-11 has high sequence homology with both human CASP4 and CASP5, we assessed both of these enzymes in our assays. When PMA-primed THP-1 cells were stimulated by LPS, the mRNA expression levels of both *CASP4* and *CASP5* were increased (**Figure 6A**); however, the fold induction of *CASP5* expression was much greater. When we examined the expression of these genes at the protein level, only CASP5 expression was increased in response to LPS, as previously reported (**Figure 6B**) (49).

To examine the roles of CASP4 and CASP5 in *HMGCR* and *HMGCS1* expression in THP-1 macrophages we knocked down *CASP4* and *CASP5* using siRNAs. Though the siRNAs used specifically knocked down *CASP4* and *CASP5* expression at the mRNA level (**Figures 6C, D**), at the protein level, knockdown of either gene appeared to affect the expression level of the other (**Figure 6E**), implying that CASP4 and CASP5 may influence each other’s post-transcriptional processes (**Figure 6E**).

As expected, *CASP4* and *CASP5* knockdown decreased the LPS-mediated induction of *HMGCR* and *HMGCS1* mRNA expression (**Figures 6C, D**). We also found that *CASP5* knockdown attenuated the LPS-stimulated induction of HMGCR and HMGCS1 at the protein level (**Figure 6F**). These data demonstrate that CASP5 is indispensable for the full induction of the SREBP1 target genes *HMGCR* and *HMGCS1* in response to LPS in THP-1 macrophages. Though the strong induction of CASP5 by LPS (**Figures 6A, B**) suggests its primary involvement in the LPS-induced expression of *HMGCR* and *HMGCS1*, the apparent secondary effects of the siRNA-mediated knockdown did not allow us to definitively determine the predominant role of CASP5 in these experiments.

## Discussion

In the present study, we have shown that caspase-11 is important for the activation of SREBP1 in response to LPS in mouse BMDMs by regulating S1P activity. After LPS stimulation, we observed that caspase-11 expression was induced, and the protein accumulated in the Golgi where it directly interacted with S1P and promoted its processing. Moreover, *Casp4* deletion resulted in reduced nuclear translocation of SREBP1 in response to LPS, and deletions of *Casp4* and *Srebf1* had similar impacts on gene expression in LPS-treated BMDMs, suggesting that caspase-11 is required for the activation of SREBP1 in response to LPS. Collectively, we have identified a novel pathway for activating SREBP1 that responds to LPS.

Our data has demonstrated that caspase-11 recognizes and directly interacts with domains 1 and 3 of S1P. In addition, we found that caspase-11 cleaves S1P at the ^694^LLVD^697^ caspase recognition site in domain 3 to generate a 50 kD fragment. Of note, production of the enzymatically active 98 kD fragment, in addition to the 50 kD fragment, was decreased in cells expressing the mouse S1P L694A/D697E mutant (**Figure 4B**). This observation suggests that caspase-11-binding site may enhance other steps of the processing of S1P, though the precise mechanisms are unknown. Moreover, the functional role of the newly identified 50 kD fragment will need to be elucidated in future studies.

Our data showing that the levels of both active S1P (98 kD; **Figure 2E**) and nuclear SREBP1 (**Figure 1C**) were decreased in LPS-treated *Casp4* ^-/-^ cells support the notion that the caspase-11/S1P axis plays an important role in the activation of SREBP1 in response to LPS. We found that SREBP1 was translocated into the nucleus 6 h after treatment with LPS in WT BMDMs (**Figure 1C**), indicating that SREBP1 is activated in the early response to LPS. Indeed, *Srebf1* deletion altered gene expression in LPS-treated BMDMs, with the expression of many LPS-induced and -repressed genes, including many genes related to inflammatory functions but unrelated to lipid metabolism, being modulated (**Figure 5B**). In agreement with these findings, previous studies have shown that SREBP1 is required for the regulation of inflammation-related genes, such as *Nlrp3* (50). These findings suggest that SREBP1 plays a pivotal role in regulating genes involved in innate immune activation in macrophages. Interestingly, the lack of SREBP1 resulted in both the upregulation and downregulation of LPS-responsive genes. SREBP1 may directly transactivate target genes or it may also directly or indirectly repress genes (10, 51). Thus, it will be important to further analyze the precise molecular mechanism of SREBP1-dependent gene repression and its role in inflammatory programs in macrophages.

In *Casp4* ^-/-^ BMDMs, the translocation of SREBP1 was reduced (**Figure 1C**), and, as expected, *Casp4* deletion affected a majority of the genes that were affected by *Srebf1* deletion (**Figure 5C**). Collectively, our data suggest that the caspase-11/S1P/SREBP1 axis is important for regulating genes involved in the innate immune activation program of macrophages. However, LPS-induced nuclear translocation of SREBP1 was not completely eliminated by *Casp4* deletion (**Figure 1C**). Previous studies have shown that the PI3K/AKT/mTOR/S6K pathway promotes the processing of SREBP1 in response to LPS; however, we found that caspase-11 was activated independently of this pathway (**Supplemental Figure 6**). Collectively, these results suggest that both the caspase-11 and PI3K/AKT/mTOR/S6K pathways may converge on SREBP1 activation during the inflammatory response. Therefore, it would be interesting to elucidate the differential and cooperative roles of these two pathways during inflammation.

In the present study, we assessed the role of the caspase-11/S1P/SREBP1 axis in the acute inflammatory response to LPS. Previous studies have mainly focused on the roles of caspase-11 in IL-1β secretion and pyroptosis and used LPS, along with transfection reagents or bacterial outer membrane vesicles that can introduce LPS into cells, to activate those functions (52, 53). In contrast, we treated BMDMs with LPS under conditions that did not induce IL-1β secretion (54). Indeed, as previously reported (55), LPS alone was not sufficient to stimulate IL-1β protein secretion from THP-1 cells (**Supplemental Figure 9**). Under these conditions, we observed that caspase-11 is activated and that it is required for S1P/SREBP1 activation. Therefore, it will be important to elucidate precisely how caspase-11 is activated in this setting of acute LPS treatment.

Though in the present study we analyzed the caspase-11/S1P/SREBP1 pathway in the acute inflammatory response, this pathway may also be important for the regulation of macrophages in chronic inflammatory pathologies, such as atherosclerosis. Interestingly, a previous study showed that in hepatocytes, caspase-2 activated SREBP2 by cleaving S1P and that genetic and pharmacologic inhibition of caspase-2 prevented liver steatosis and non-alcoholic steatohepatitis, which are caused by chronic inflammation, by suppressing SREBP2 activation (46). Moreover, SREBP1-mediated lipid synthesis was shown to contribute to phagocytic activity as well as the late suppression of inflammatory activation, which are crucial for the proper resolution of inflammation (9). As such, further studies are needed to elucidate how the caspase-11/S1P/SREBP1 pathway regulates lipid metabolism and how this regulation is linked to macrophage activities, particularly in the context of chronic inflammation.

In summary, we have identified a novel caspase-11/S1P/SREBP1 pathway that may act as a key immunometabolic regulator of macrophage immune responses.

## Materials and methods

### Antibodies and reagents

The anti-FLAG and anti-caspase-11 antibodies were from Sigma. The anti-SREBP1, anti-HMGCS1, anti-HMGCR antibodies were from Santa Cruz Biotechnology. The anti-HA antibody was from Roche, and the anti-S1P and anti-GAPDH antibodies were from Abcam. The anti-RCAS1, anti-EEA1, anti-LAMP1, anti-human caspase-4 and anti-caspase-5 antibodies were from Cell Signaling Technology. The LPS was purchased from Sigma (L2637).

### Animals

*Casp4^-/-^* mice were provided by Dr. Masayuki Miura (Graduate School of Pharmaceutical Sciences, The University of Tokyo). *Srebf1^-/-^* mice were provided by Dr. Hitoshi Shimano (The University of Tsukuba). All of the mice used in this study were on the C57BL/6 background. All mice were maintained in the institutional animal house facility with a 12 h/12 h light-dark cycle and free access to food and water. All experimental procedures were performed in accordance with the research guidelines for the care and use of laboratory animals of Nippon Medical School.

### Culture of bone marrow-derived macrophages (BMDMs)

Femurs and tibias were collected from the hind legs of *Casp4^-/-^*, *Srebf1^-/-^*, or wild-type control mice at 8 to 12 weeks of age. Muscles attached to the bones were removed using clean and sterile scissors and forceps. Marrow was flushed from the bones using a syringe filled with RPMI 1640 medium and collected in a sterile 50 ml tube. After centrifugation at 500 x *g* for 5 min, cells were resuspended in RPMI 1640 medium supplemented with 10% fetal bovine serum (FBS), 20 ng/mL M-CSF (Biolegend), 100 U/ml penicillin, and 100 mg/ml streptomycin (growth medium). Subsequently, the cells were plated on 150 mm non-tissue culture-treated plates (FALCON) at a density of 4–6 x 10^6^ cells/plate and incubated at 37˚C in a 5% CO_2_ atmosphere. On the sixth day, supernatants were discarded, and the differentiated cells were collected. Finally, the BMDMs were counted and seeded onto tissue culture plates for 24 h before performing experimental procedures, during which, the culture medium was replaced with fresh growth medium. LPS (100 ng/ml) was added to the culture medium to stimulate cells.

### Cell culture

HEK293T cells (ATCC) were cultured in DMEM (Nakarai Tesque) supplemented with 10% heat-inactivated FBS (Hyclone, GE Healthcare), 100 U/ml penicillin, and 100 mg/ml streptomycin. The human monocytic cell line THP-1 (ATCC TIB-202) was maintained in RPMI 1640 medium supplemented with 10% FBS, 100 U/ml penicillin and 100 mg/ml streptomycin. Cells were seeded at 2 x 10^5^ cells per well in a 24-well plate. The cells were then differentiated to acquire macrophage-like morphology by incubating them with 5 x 10^-8^ M phorbol 12-myristate 13-acetate (PMA) (Sigma-Aldrich) for 72 h in culture. The differentiated THP-1 cells were then cultured in fresh RPMI 1640 medium containing 10% FBS (Invitrogen) and washed three times with medium before siRNA transfection. All the cells used in these experiments were cultured at 37˚C in a 5% CO_2_ atmosphere.

### siRNA transfection

siRNAs were transiently transfected using Lipofectamine RNAiMAX Transfection Reagent (Thermo Fisher Scientific) according to the manufacturer’s protocol. Forty-eight hours after transfection, cells were harvested and subjected to RT-PCR analysis.

### RNA isolation and RT-PCR

RNA was isolated from cultured cells using ISOGEN reagent (NIPPON GENE). cDNA was prepared using RT Master Mix with gDNA Remover (TOYOBO), according to the manufacturer’s instructions. Real-time quantitative PCR (qPCR) was performed on cDNA (25 ng) using a Step One Plus Real-Time PCR System and Power-Up SYBR Green Master Mix (Applied Biosystems). The primer sequences used in this study are shown in **Supplemental Table 1**. *Gapdh* expression was used as an internal control.

### Construction of plasmids

The expression plasmids for S1P and caspase-11 were constructed by PCR amplification using wild-type mouse BMDM cDNA as a template. The amplified inserts were then ligated into either pCMV-3HA or pCMV-3FLAG empty vectors using the Rapid DNA Dephos & Ligation Kit (Roche).

### Transfection and detection of S1P

HEK293T cells (2.5 x 10^5^ cells, cultured in 10 cm dishes) were transfected with expression plasmids (2.0 μg in total) using FuGENE^®^ HD Transfection Reagent (Promega), according to the manufacturer’s instructions. Twenty-four hours post-transfection, the cells were washed, then collected and lysed in 1X lysis buffer (50 mM NaCl; 200 mM Tris-HCl, pH 7.5; 1 mM EDTA; 1% NP40). To detect secreted S1P in culture medium, cells were plated at a density of 5 x 10^5^ cells per well in 6-well plates. On the next day, 1.5 μg of the indicated DNA vectors were transfected into the cells using FuGENE^®^ HD Transfection Reagent, according to the manufacturer’s instructions. After 5 h, the cells were incubated in DMEM medium supplemented with 1% lipoprotein-deficient serum. After 24 hours, whole-cell lysates were prepared by lysing cells in 1X lysis buffer (50 mM NaCl; 200 mM Tris-HCl, pH 7.5; 1 mM EDTA; 1% NP-40), sonicating 3 times (30 s on and 30 s off), and boiling at 95˚C for 5 min before western blot analysis. Culture supernatants were mixed with Total Exosome Isolation Reagent (Thermo Fisher Scientific, Cat. No. 4478359) and incubated at 4˚C overnight. According to the manufacturer’s instructions, the culture medium was centrifugated at 10000 x *g* for 1 hour, then the pellets were dissolved in sample buffer and run on an SDS-PAGE gel.

### Western blotting

Cells were collected and lysed in lysis buffer (50 mM NaCl; 200 mM Tris-HCl, pH 7.5; 1 mM EDTA; 1% NP-40 and Protease Inhibitors). Lysates were then boiled at 95˚C for 5 min, separated by 8 or 10% SDS-PAGE, and then transferred onto PVDF membranes. After blocking for 1 h at room temperature with blocking buffer (5% BSA, TBS + 0.1% Triton X-100), membranes were incubated with primary antibody at 4˚C overnight, followed by incubation with the appropriate secondary antibodies for 1 h. Protein bands were detected using chemiluminescent (ECL) substrate (GE Healthcare). To collect nuclear proteins, cells were harvested and lysed in lysis buffer (50 mM NaCl; 200 mM Tris-HCl, pH 7.5; 1 mM EDTA; 1% NP-40 and Protease Inhibitors) with 0.1 M DTT and centrifuged at 11000 x g for 1 min. The pellet was then resuspended in extraction buffer (20 mM HEPES, pH 7.9; 1.5 mM MgCl2; 0.6 mM EDTA, 30 mM KCL and 75% (v/v) glycerol), sonicated for 5 seconds, and centrifuged at 20000 x g for 5 min.

### Immunoprecipitation

Cells were collected in lysis buffer (50 mM NaCl; 200 mM Tris-HCl, pH 7.5; 1 mM EDTA; 1% NP-40 and Protease Inhibitors). Lysates were precipitated with antibody at 4˚C overnight, then protein G-agarose beads (GE Healthcare) were added, and the samples were incubated for 1 h at 4˚C. The beads were washed five times with lysis buffer, and bound proteins were removed by boiling in SDS sample buffer and resolved on a 10% SDS-PAGE gel.

### Immunofluorescence staining

BMDMs were grown on 4‐well culture slides (FALCON) for 24 h and fixed with 4% paraformaldehyde for 15 min. After fixation, cells were washed three times with PBS, permeabilized with 0.1% Triton X‐100 for 15 min, and blocked with 2% BSA/PBS for 1 h. The slides were then incubated overnight with primary antibodies (1:1000) at 4˚C, and after thorough washing, were incubated with secondary antibody (1:10000, Life Technologies). The slides were mounted with 4’,6‐diamidino‐2‐phenylindole (DAPI)‐containing mounting medium (Vector Laboratories), and images were acquired with a confocal microscope (LSM 710 META, Carl Zeiss). Obtained images were processed using the Zeiss LSM Image Browser.

### RNA sequencing

Total RNA was isolated and purified using the RNeasy Mini Kit, according to the manufacturer’s instructions (QIAGEN). Poly-A mRNA was extracted from the total RNA using oligo-dT beads in a NEBNext Poly (A) RNA Magnetic Isolation Module (New England Biolabs), after which, RNA-seq libraries were prepared using the RNA-Seq library preparation kit for Illumina (New England Biolabs), according to the manufacturer’s protocols. Libraries were paired-end-sequenced on a NovaSeq sequencer (Illumina). Reads were aligned with the mm10 mouse genome using STAR (56). Aligned read files were analyzed using HOMER (57). Batch effects were removed using ComBat-seq (58). Differentially expressed genes were analyzed using DESeq2 (59). For clustering, standardized rlog values computed by DESeq2 were used. Hierarchical clustering was performed using the complete linkage method of Euclidean distance by cluster 3.0 (60). Enrichment analysis was performed using Metascape (48) with the default parameters.

### Statistics and reproducibility

Sample sizes were not based on power calculations. Data are presented as means ± SEM, except where otherwise indicated. For experiments involving two factors, data were analyzed using two-way ANOVA followed by Tukey’s tests, except where otherwise indicated. Values of *P* < 0.05 were considered significant.

## Data availability statement

The datasets presented in this study can be found in online repositories. The names of the repository/repositories and accession number(s) can be found below: GEO under accession number GSE208374.

## Ethics statement

The animal study was reviewed and approved by Animal Experiment Committee, Nippon Medical School.

## Author contributions

YC, IM, SH, YE, and YO carried out the experiments and analyzed the data. YC, YO, and IM wrote the manuscript. YO conceived the original idea and supervised the project. All authors contributed to the article and approved the submitted version.
