# Supplementary material for: Caspase-11 contributes to site-1 protease cleavage and SREBP1 activation in the inflammatory response of macrophages
Source: Front Immunol. 2023 Jan 27;14:1009973. doi: 10.3389/fimmu.2023.1009973 (PMC9912839; doi:10.3389/fimmu.2023.1009973)
Supplement: Supplementary file 1 [file DataSheet_1.pdf]

Materials and methods for Supplemental Information

Antibodies and reagents

The anti-phospho S6K, -phospho S6, -S6K, and -S6 antibodies were from Cell Signaling Technology. The anti-caspase-1, and anti-caspase-12 antibodies were from Santa Cruz Biotechnology. The LY293002 and Rapamycin were from Cell Signaling Technology.

IL-1β ELISA

Culture medium was collected from THP-1 cells and analyzed using a human IL-1β ELISA kit (R&D) according to the manufacturer's instructions (n=3).

Supplemental Figure Legends

Supplemental Figure 1. Caspase-1 and -12 expression in BMDMs

(A) *Casp1* and *Casp12* mRNA expression in BMDMs treated with 100 ng/ml LPS for 0, 2, 4 and 6 h is shown. Error bars represent means ± SEM. Significance was determined by one-way ANOVA followed by Tukey's multiple comparison test. $*P < 0.05$, $**P < 0.01$.

(B) BMDMs were treated with LPS for the indicated times. Whole cell lysates were collected, and the expressions of caspase-1 and -12 proteins were analyzed by western blot. It is worth noting that bands corresponding to pro-caspase-1 (45kD) and pro-caspase-12 (42kD) were detected, while bands corresponding to intermediate caspase-1 (35kD), active caspase-1 (30kD), and active caspase-12 (38kD) were not detected.

Supplemental Figure 2. *Srebf1a* expression in *Casp4*$^{-/-}$ BMDMs

Srebf1a mRNA expression levels in WT and *Casp4*$^{-/-}$ BMDMs treated with LPS for the indicated times were determined by qPCR. *Srebf1a* mRNA levels were first normalized to those of *Gapdh* and then to the level of *Srebf1a* expression in untreated, WT BMDMs. Data represent means ± SEM. Significance was determined by one-way

ANOVA followed by Tukey's multiple comparison test. *n*=3. **P* < 0.05 vs. untreated cells of the same genotype. ns: not significant.

Supplemental Figure 3. Subcellular localization of caspase-11

(A and B) BMDMs were stimulated with LPS for the indicated times. Caspase-11 (green), S1P (red), the ER marker PDI (A, light blue), and the lysosome marker LAMP1 (B, light blue) were visualized by immunofluorescence staining. Nuclei were counterstained with DAPI (blue). Scale bars, 10 μm.

Supplemental Figure 4. Expression of *Mbtps1* in LPS-treated BMDMs

Mbtps1 mRNA expression levels in WT and $Casp4^{-/-}$ BMDMs treated with LPS for the indicated times were determined by qPCR. *Mbtps1* mRNA levels were first normalized to those of *Gapdh* and then to the level of *Mbtps1* expression in untreated, WT BMDMs. Data represent means ± SEM. Significance was determined by one-way ANOVA followed by Tukey's multiple comparison test. *n*=3 for each time point. **P* < 0.05 vs. untreated cells of the same genotype. ns: not significant.

Supplemental Figure 5. Caspase-11 is activated independently of SCAP and S2P

(A) WT BMDMs were first transfected with *Scap*, *Mbtps2* (S2P), or control (siCont) siRNAs. Forty-eight hours after transfection, the mRNA expression levels of *Scap* and *Mbtps2* were analyzed by qPCR. Significance was determined using unpaired Student's *t*-tests. **P* < 0.05. *n*=3 for each group.

(B) WT BMDMs were transfected with control, *Scap*, or *Mbtps2* siRNAs and then stimulated with or without LPS (100 ng/ml) for 6 h. Caspase-11 proteins were analyzed by western blot. GAPDH expression is shown as a loading control.

(C) *Scap* and *Mbtps2* mRNA expression levels in untreated WT and $Casp4^{-/-}$ BMDMs were analyzed by qPCR. Unpaired Student's *t*-tests were used to determine significance. ns: not significant. *n*=3 for each group.

Supplemental Figure 6. Activation of the caspase-11 and AKT/mTOR signaling pathways

(A and B) WT and $Casp4^{-/-}$ BMDMs were treated with rapamycin (A, 100 nM) or LY294002 (B, 10 μM) for 1 h, followed by LPS (100 ng/ml) for another 6 h, as indicated. Whole cell lysates were collected and subjected to western blot analysis to examine the levels of phosphorylated and total S6K and S6 proteins. GAPDH expression is shown as a loading control.

(C and D) The protein expression levels of caspase-11 in WT BMDMs pretreated with rapamycin (C) or LY294002 (D) followed by 6 h of LPS stimulation were determined by western blot analysis. Under these electrophoresis conditions, the pro-caspase-11 did not separate into its 38 kD and 40 kD bands.

Supplemental Figure 7. Conservation of the caspase-11 cleavage site in S1P

Alignment of the S1P (MBTPS1) amino acid sequences of multiple species around the predicted caspase-11 cleavage site. Residue numbers are for the mouse amino acid sequence (NC_000074.7). Human, NC_000016.10; rat, NC_005118.4; hamster NC_048596.1.

Supplemental Figure 8. SREBP1 binding to the *Hmgcs1* and *Hmgcr* genes

UCSC genome browser images illustrating the normalized tag counts for H3K27Ac and SREBP1 at the *Hmgcs1* (A) and *Hmgcr* (B) gene loci in WT and *Srebf*$^{-/-}$ BMDMs left untreated (KLA 0 h) or treated with KLA (Kdo2-lipid A, a TLR4 agonist) for 6 h (KLA 6 h). Note that the numbers of H3K27Ac tags around the *Hmgcs1* and *Hmgcr* enhancers were increased after 6 h of TLR4 activation.

Supplemental Figure 9. IL-1β protein secretion from THP-1 macrophages

THP-1 cells were stimulated with LPS, ATP, or a combination of the two for 6 h, as indicated. Samples of the culture media were collected and IL-1β protein levels were quantified by ELISA.

Supplemental Table 1. The primers used in this study

	Forward	Reverse
Gapdh	AATGTGTCCGTCGTGGATCT	CATCGAAGGTGGAAGAGTGG
Casp1	ACAAGGCACGGGACCTATG	TCCCAGTCAGTCCTGGAAATG
Casp4	ACAAACACCCTGACAAACCAC	CACTGCGTTCAGCATTGTTAAA
Casp12	AGACAGAGTTAATGCAGTTTGCT	TCCACCCCACAGATTCCTTCC
Hmgcr	AATGCCTTGTGATTGGAGTTG	TGGCCAACACTGACATGC
Hmgcs1	CAGGGTCTGATCCCCTTTGG	CAGAGAACTGTGGTCTCCAGGT
Mbtps1	CTGGTGGTTTTGCTCTGTGG	GGCTGTGAAGTATCCGTTGAAAG
Mbtps2	GTACCGTCCTTGGAAACTCAC	CACTGTGTAGTGAAGATGCAGTGGG
SCAP	GGTGAAGCTTCGGATTGCAC	CAGGCACGAGGGTGAAGTAG
GAPDH	GGAGCGAGATCCCTCCAAAAT	GGCTGTTGTCATACTTCTCATGG
CASP4	CAAGAGAAGCAACGTATGGCA	AGGCAGATGGTCAAACTCTGTA
CASP5	CCGGATGTGCTGCTTTATGAC	GCAAACAGAATCTGCCTCCA
HMGCR	TGATTGACCTTTCCAGAGCAAG	CTAAAATTGCCATTCCACGAGC
HMGCS1	GATGTGGGAATTGTTGCCCTT	ATTGTCTCTGTTCCAACTTCCAG
Srebf1a	GGCCGAGATGTGCGAACT	TTGTTGATGAGCTGGAGCATGT

Supplemental Table 2.

LPS-induced genes

Cluster 1

Tiam1, *Htt*, *Cemip2*, *Itpr1*, *Aff1*, *Apaf1*, *Pikfyve*, *Abl2*, *Tgs1*, *Atp11b*, *Eif4g3*, *Dcp1a*, *Avl9*, *Jarid2*, *Cdyl2*, *Spta1*, *Arid5b*, *Ddx60*, *Oas2*, *Zfp800*, *Hivep2*, *Elmod2*, *Atxn7l1*, *Dgka*, *Fgd6*, *Slfn4*, *Tanc1*, *Rapgef2*, *Chd9*

Cluster 2

Cwf19l2, *Homer1*, *Rufy3*, *Igf2bp2*, *Hmgcs1*, *Dennd6b*, *Fgl2*, *Inppl1*, *Tnfrsf11a*, *Adap2*, *Egr1*, *Zscan29*, *Dr1*, *Tubb6*, *Cst7*, *Qpct*, *Pla2r1*, *BC147527*, *Cdyl*, *Syne3*, *Tnks1bp1*, *Vma21*, *Ppp1r10*, *Parp3*, *Jak3*, *Fbrsl1*, *Slc12a9*, *Gnas*, *Esyt1*, *Akt2*, *Upp1*, *Sdc3*, *Socs1*, *Gns*, *Lhx2*, *Cndp2*, *Ctsa*, *Adam8*, *Cd68*, *Trem2*, *Gpnmb*, *Themis2*, *Ms4a4c*, *Pltp*, *Irf8*, *Lgmn*, *Fcgr1*, *Folr2*, *Ccl7*, *Rgs1*, *Arid5a*, *Edn1*, *Ccl2*, *Cd72*, *Hmga1*, *Hmga1b*, *Ccl6*, *Ckb*, *Aldh1b1*, *Dusp1*, *Prnp*, *Serpina3g*, *Ifit1bl1*, *Pcna*, *Bzw2*, *Tpx2*, *Hk3*, *Ifi214*, *Tmpo*, *Naaa*, *Odc1*, *Klrk1*, *Diaph1*, *Ccr3*, *Bst1*, *Map2k3*, *Phyh*, *Il18rap*, *Ptger4*, *Zfp777*, *Casp3*, *Nkain1*, *C1ra*, *Hbegf*, *Tagap*, *Sgcb*, *Glipr2*, *Il4ra*, *Lipg*, *Zyx*, *Ralgds*, *Rmdn3*, *Mgat1*, *Pacs1*, *Mppe1*, *Kmo*, *Aldh1l1*, *Armcx6*, *Serpina3f*, *Cnn3*, *Mxd1*, *Ccl8*, *Sertad3*, *Carhsp1*, *Zbtb8a*, *Batf2*, *C3ar1*, *Il10*, *Lck*, *Cxcl9*, *Ifit3b*, *Rab43*, *Fcgr4*, *Rhoh*, *Gadd45g*, *B430306N03Rik*, *Cxcl11*, *Cdkn2d*, *Fbxo42*, *Tgtp1*, *Furin*, *Dek*, *Plod1*, *Dnaaf3*, *Ndst2*, *Lrrc4*, *Marc2*, *Tpst2*, *Plbd2*

Cluster 3

Hcar2, *Batf*, *H2-M2*, *Casp4*, *Tlr2*, *Lmo4*, *Cd82*, *Fpr1*, *Mefv*, *Sqle*, *Nab2*, *Pla2g7*, *Plac8*, *H2-Ab1*, *C130026I21Rik*, *Bend6*, *H2-Aa*, *H2-Eb1*, *Gpr18*

Cluster 4

Sass6, *Btk*, *Rnf125*, *Tmigd3*, *Adora3*, *Snap29*, *Gm12185*, *I830077J02Rik*, *Rubcnl*, *S100a9*, *Nemp1*, *Brdt*, *Gm5431*, *Rin2*, *Gm6377*, *Gm4951*, *Iigp1*, *Tcf4*, *Tiparp*, *Coro2a*, *Ifi208*, *Ripk1*, *Scimp*, *Gem*, *Lrch1*, *Bcl6*, *Elf1*, *Ifi213*, *Gbp4*, *Nos2*, *Phf11d*, *Tgtp2*, *AW112010*, *Fcgr3*, *Phf11a*, *Apoe*, *Selenop*, *5430427O19Rik*, *Il13ra1*, *Cd200r4*, *Ccr5*, *Fas*, *Slamf8*, *Cr1l*, *Tmem171*, *Phf11b*, *H2-T22*, *H2-T9*, *Zbp1*, *Ccl12*, *H2-T23*, *C1qb*, *Mmp13*, *Ms4a6d*, *C1qc*, *Hexb*, *Calhm6*, *Noc4l*, *Cited2*, *Klf6*, *Cd274*, *Ifi209*, *Ifit3*, *Cd69*, *Cd53*, *Cd83*, *Pou3f1*, *Herpud2*, *Otud1*, *Tmem243*, *Arf6*, *B3gnt2*, *Lyn*, *Brd2*, *Dhx58*, *Arl4a*, *Abhd16a*, *Trip10*, *Nod1*, *Inpp5b*, *C5ar1*, *Plaur*, *Stom*, *Irf5*, *Tgm2*, *Rela*, *Sertad1*, *Kcnn4*, *Mafk*, *Os9*, *Procr*, *Rbms1*, *Spred1*, *4931440P22Rik*, *Tor1aip2*, *Rab8b*, *Slc6a6*, *Ppp1r15b*, *Prkx*, *Chd7*, *Bcl9*, *Ogfrl1*, *Gbp9*, *Zcchc2*, *Nfxl1*, *Dck*, *Mcmbp*, *Plekhf2*, *Cbfb*, *Pml*, *Adam9*, *Sco1*, *Il15ra*, *Plekha2*, *Mtus1*, *Map2k4*, *Acvr1*, *Nes*, *Vezt*, *Ncoa7*, *Slc28a2*, *Tnfsf10*, *Mcur1*, *Tulp3*, *Xdh*, *Rap1gds1*, *Abtb2*, *Slc12a7*, *Cmpk2*, *Rsad2*, *Ifit2*, *Mx1*, *Cd36*, *Sh3bgrl*, *Cd164*, *Slamf7*, *Parp11*

Cluster 5

Selp, *Dpysl2*, *Ugcg*, *Slfn1*, *Il18bp*, *Ccr1*, *Morf4l1*, *Ly6a*, *Vav1*, *Mul1*, *Litaf*, *Cfp*, *C1qa*, *Man2b1*, *P2rx4*, *Cyren*, *Tmem140*, *1600014C10Rik*, *Smpdl3a*, *Slamf9*, *Cxcl16*, *Nupr1*, *A530032D15Rik*, *Aif1*, *Pf4*, *Glrx*, *Egln2*, *Agpat2*, *Tesk1*, *Limd2*, *Ppm1g*, *Lpcat2*, *H2-Q8*, *H2-Q6*, *Cmtm7*, *H2-Q9*, *H2-Q7*, *AB124611*, *Ly86*, *Ecm1*, *Cnp*, *Ptpn1*, *Slc9a3r1*, *Flot1*, *Nrbp1*, *Frmd8*, *Ube2r2*, *St6galnac4*, *Ltbr*, *Itpk1*, *Tmed1*, *Igf2bp3*, *Zfand3*, *Ch25h*, *Cpq*, *Itga9*, *Ppp1r13b*, *Myo1c*, *Jun*, *Git1*, *Fez2*, *Hmgn3*, *Samsn1*, *Crk*, *Gbp6*, *Ms4a6b*,

Tmod3, *Cyb5r4*, *Xaf1*, *Plekhb2*, *Nkiras2*, *Zfp385c*, *F2r*, *Ctsh*, *Grap*, *Mmp12*, *Zmynd15*, *Ms4a7*, *Bcl2a1b*, *Cd74*, *Tank*, *Ifi44*, *H2-T10*, *Irf2*, *Sema4c*, *Plcl2*, *Phc2*, *Foxp4*, *Tent5a*, *Ggta1*, *March1*, *Man1a*, *Spata13*, *Rhbdf1*, *Mmd*, *Itprip*, *H2-Q5*, *S100a8*, *Aoah*, *Spib*, *Apol7c*, *Necap1*, *Gm18852*, *Gm18853*

Cluster 6

Sh3bp5, *Trip13*, *Flvcr2*, *Il20rb*, *Gatd3a*, *Mfap3l*, *Abce1*, *Arhgap6*, *Zfp324*, *Fubp1*, *Rrs1*, *Bmp1*, *Stk38l*, *Pdgfrb*, *Micall2*, *Slc16a10*, *Ikbkb*, *Cct6a*, *Tbc1d15*, *Tnfsf15*, *Nxpe3*, *Raet1c*, *Trpm4*, *Stim2*, *Txnrd1*, *Ppfia3*, *Gstm1*, *Nup62*, *Cx3cl1*, *Pvr*, *Ak4*, *Rab32*, *Axl*, *Il6*, *Spint2*, *Tnfsf9*, *Adora2a*, *Il4i1*, *Il4i1b*, *Rhob*, *Atg9a*, *Hp*, *Ptafr*, *Nop16*, *Sema4a*, *Fscn1*, *Kdm4a*, *F11r*, *Plat*, *Atf5*, *Alas1*, *Fam98a*, *Mybbp1a*, *Cxcl3*, *Ccl22*, *Hmox1*, *Prep*, *Sdc1*, *Cacnb3*, *Rnd1*, *Tars*, *Col4a2*, *Ubxn2a*, *Lad1*, *Spcs3*, *Ptges*, *Gfi1*, *Nsun2*, *Pde2a*, *Ppic*, *Txndc5*, *Herpud1*, *Wdr36*, *Pa2g4*, *Tspyl3*, *Cyb5b*, *Ido2*, *Ccl17*, *Rrp12*, *H60b*, *Emp2*, *Gars*, *Rras2*, *Vgf*, *Ptx3*, *1110032F04Rik*, *Abcb6*, *Il1f9*, *Cacng8*, *Epop*, *Manf*, *Pdia6*, *Gpc1*, *Oaf*, *Jdp2*, *Iars*, *Cav1*, *Osbpl3*, *Blmh*, *Uchl1*, *Cav2*, *Asns*, *Aldh18a1*, *Cars*, *Rpn1*, *Col18a1*, *Dner*, *Ccl24*, *Eda2r*, *Mpp6*, *Slc13a3*, *Macc1*, *Cuedc1*, *Tuba4a*, *Bahcc1*, *Ddhd1*, *Hdc*, *Zdhhc2*, *Shisa3*, *Me1*, *Col4a1*, *Hyou1*, *Synpo2*, *Gm23450*, *Mgst2*, *Grhl1*, *Dtnb*, *Hs3st3b1*, *Itsn2*, *Mdn1*, *Ube3a*, *Tnc*, *Cobll1*, *Atp10a*, *Arhgap35*, *Cdc42bpa*, *Utp20*, *Hinfp*, *Dnajc3*, *Tmx3*, *Eprs*, *Il1bos*, *Diaph3*, *Lztfl1*, *Rtn1*, *Trim36*, *Met*, *Appl1*, *Dnm1l*, *Yme1l1*, *9330159M07Rik*, *Dcbld2*, *Gpr85*, *Jpt2*, *Insig1*, *Col27a1*, *Pspc1*, *Ppp4r2*, *Sept11*, *Ell2*, *Zeb1*, *Malt1*, *Fam49a*, *Dpep3*, *Lnpk*, *Bicd2*, *Ipo7*, *Tshz1*, *Srp72*, *Dsel*, *Abcc4*, *Far1*, *Akap2*, *Pakap*, *Ube4a*, *Zfp422*, *Lox*, *Rgs16*, *Fhod3*, *Grk3*, *Tbc1d23*, *Ibtk*, *Gigyf2*, *Tardbp*, *Ago1*, *Stard4*, *Fasn*, *Sec23a*, *Slc38a2*, *Dhx40*, *Adamts4*, *Has1*, *Ikbkg*, *Nup160*, *Ralgapa2*, *Ythdc2*, *Itgb8*, *Senp6*, *Dot1l*, *Tacc1*, *Slc4a7*, *Ppip5k2*, *Pank3*, *Dock10*, *Atp11c*, *Arhgap32*, *Gpd2*, *Ptch1*, *Kidins220*, *Daam1*, *Trim56*, *Ireb2*, *Trpm7*, *Mki67*, *Atad2b*, *Lrrc58*, *Otud4*, *Magt1*, *Cntrl*, *Myo10*, *Edem1*, *Ago3*, *Hivep3*, *Zc3h12c*, *Il17rd*, *Mapkbp1*, *Rif1*, *Xpo4*, *Taf1*, *Abcc5*, *Ccnt1*, *Rest*, *Zfp26*, *Strn*, *Larp4*, *Bms1*, *Heg1*, *Flnb*, *Gfpt1*, *Huwe1*

Cluster 7

Zdhhc20, *Speccl1*, *Eif5*, *Pogz*, *Rhoq*, *Shoc2*, *Ppp1cb*, *Dusp16*, *Rnf24*, *Ankrd33b*, *Rasgrp1*, *Sh3bgrl2*, *Syncrip*, *Pde4b*, *Ube2w*, *Baz1a*, *Mir22hg*, *Rlf*, *Eea1*, *Wdr59*, *Hspa4l*, *Zfp407*, *Usp34*, *Zfp263*, *Foxn2*, *Clock*, *Zhx1*, *Ero1l*, *Hspd1*, *Kpna1*, *Phtf2*, *Ddx21*, *Hspa13*, *Adgrl2*, *Abcc1*, *Prdm1*, *Atp2b4*, *Rab11fip1*, *Dennd4a*, *Ccdc88b*, *Hmgcr*, *Uso1*, *Rasa2*, *Agrn*, *Heca*, *Klf7*, *Creb1*, *Tsr1*, *Phldb1*, *Ipo5*, *E2f8*, *Rhou*, *Abhd2*, *Pop1*, *Cysltr1*, *Nr4a3*, *Sgms1*, *Slc25a37*, *6530402F18Rik*, *Flrt3*, *Palld*, *Kdm6b*, *Cnbp*, *Slc6a12*, *Ifrd1*, *Rin3*, *Stard5*, *Usp10*, *Slc2a1*, *Ift57*, *Cxcl2*, *Ets2*, *Clec5a*, *Slc39a14*, *Ampd3*, *Rcsd1*, *Fam114a1*, *Hcfc2*, *Tirap*, *Orai2*, *Snx18*, *Reps1*, *Akna*, *Icosl*, *St3gal1*, *Rapgef1*, *Acvr1b*, *Slc7a11*, *Asap1*, *Sde2*, *Sla*, *Arfgap3*, *Pls3*, *Rhof*, *Ptgir*, *Marco*, *Tmem120b*, *Ier3*, *Ifi27*, *Slc16a3*, *Id3*, *Gdf15*, *Uba5*, *St3gal3*, *Snx25*, *Ufm1*, *Gm13889*, *Pfkl*, *AI662270*, *Ass1*, *Gm5424*, *Slc39a7*, *Atf6b*, *Rdh13*, *Fam107b*, *Ggps1*, *Bnip3*, *Raet1e*, *Ppfibp2*, *Itgal*, *Polr3d*, *Trib1*, *Irak3*, *Kmt5a*, *Ccne1*, *Tmem185b*, *Il12b*, *Wdr5*, *Nfe2l2*, *Msantd4*, *Rrn3*, *Ticam2*, *Mat2a*, *Cxcl1*, *Jmjd6*, *Il12a*, *Lcn2*, *Fam20c*, *Tspan33*, *Tmem176a*, *Ttc39c*, *Top1*, *B3galt6*, *Amd1*, *Amd2*, *Stxbp6*, *Serpinb2*, *Pim3*

Cluster 8

Slc30a1, *Nox1*, *Bcl2a1a*, *Raet1b*, *Raet1a*, *Raet1d*, *Plxdc2*, *Mycl*, *B3gnt7*, *C920009B18Rik*, *Cul4a*, *Hist3h2a*, *Aqp9*, *Morf4l1-ps1*, *Srebf1*, *Bckdhb*, *Adora2b*, *Tpbg*, *Aggf1*, *Fosl2*, *Atp2b1*, *Slc7a2*, *G530011O06Rik*, *Erdr1*, *Col4a5*, *Dnajb4*, *Eif2s3y*, *Ednrb*, *Tnfsf4*, *Mcm10*, *Il34*, *Nt5c1a*, *Yars*, *Mthfd2*, *Ptprn*, *Car5b*, *Aars*,

Itga1, *Sntb1*, *Chsy3*, *Slc7a1*, *Bcl2l11*, *Pde12*, *Kcnab1*, *Ncbp3*, *Gnl3l*, *Slc7a5*, *Hsp90b1*, *Tarm1*, *Lrrk2*, *Myo1d*, *Snn*, *Vcam1*, *Csf1*, *Cdk5r1*, *Cdc42ep2*, *Psat1*, *Errfi1*, *Mid1*, *Ddx19b*, *Vcan*, *Pcdh7*, *Slc30a4*, *Cdk14*

LPS-repressed genes

Cluster 1

Rnf150, *Arhgap18*, *Snx30*, *Hip1*, *St8sia4*, *Tfrc*, *Cd93*, *Zfp36l2*, *Tnrc18*, *Pcmtd1*, *Wdfy2*, *Mrc1*, *Eps8l1*, *Ttyh3*, *Sgpp1*, *Ccnd1*, *Oxct1*, *Asph*, *Dhrs9*, *BC005537*, *Igf1*, *Tmem94*, *Prune2*, *Nceh1*, *Epb41l1*, *2610507B11Rik*, *Rnasel*, *Lima1*

Cluster 2

Spr, *Erp29*, *1700037H04Rik*, *Scarb1*, *Eid1*, *Adssl1*, *Slc37a2*, *Pcp4l1*, *Atp6v0a1*, *Idh2*, *Myo1f*, *Coro1c*, *Adamts10*, *Pparg*, *Mlycd*, *Clvs1*, *Nqo2*, *Rgs2*, *Cd5l*, *Apobec1*, *Fam53a*, *Snx5*, *Slc27a1*, *Wdr62*, *Osbpl7*, *Tubb5*, *Lat2*, *Gamt*, *Arhgap9*, *Tle5*

Cluster 3

Neurl3, *Tmem51*, *Stat6*, *Osbpl9*, *Pigt*, *Arrb2*, *Tom1l2*, *Tvp23b*, *Atp2a3*, *Ifngr1*, *Mamdc2*, *Ide*, *Cep162*, *Dapk1*, *Rai1*, *Ltbp3*, *Mmgt2*, *Map3k3*, *Ypel5*, *Sulf2*, *Adipor1*, *Acap1*, *Fcgrt*, *Mindy2*, *Neo1*, *Cgnl1*, *Adgre5*, *Entpd1*, *Clec7a*

Cluster 4

Dok2, *Ubald2*, *Ifi30*, *Tbkbp1*, *Daglb*, *Mid1ip1*, *Dhrs3*, *Adam15*, *Macrod1*, *Zdhhc14*, *Cd151*, *Lmo2*, *Slc6a8*, *Sipa1*, *Mafb*, *Ypel3*, *Cgrrf1*, *Tspan4*, *Tgif1*, *Gla*, *Naglu*, *Pstpip1*, *Akirin2*, *Wtip*, *P2ry6*, *Fam234a*, *Dbndd2*, *Timp2*, *Pqlc1*, *Gas6*, *Csk*, *Mta3*, *Tnfsfm13*, *Itgb5*, *Cyp4f13*, *Pkib*, *Nme3*, *Pink1*, *Gng2*, *Syp*, *Pianp*, *Trim47*, *Glul*, *Cd300a*, *Map3k10*, *Agap3*, *Syngr1*, *Il11ra1*, *Inpp5k*, *Pagr1a*, *Tbxas1*

Cluster 5

Hmga2, *Rgs18*, *Vipas39*, *Pmp22*, *Gyg*, *2310022A10Rik*, *Tm6sf1*, *B4gat1*, *Abcg1*, *Hhex*, *Cd200r1*, *Pla2g15*, *Lpxn*, *Tmem8*, *Atp6v0d2*, *Cttnbp2nl*, *Mertk*, *Ski*, *Dab2*, *Itpkb*, *Dpy19l3*, *Tspan13*, *Mmp27*, *Pcyox1*, *Hpse*, *Cd84*, *Slco2b1*, *St6gal1*, *Hpgd*, *Lasp1*, *Elmo2*, *Rasa3*, *Ttc7*, *Acss1*, *Gask1b*, *Ripor1*, *Rab11fip5*, *Adcy3*, *Mknk2*, *Pygb*, *Evi2b*, *Smagp*, *Rnf217*, *Tec*, *B4galt6*, *Numa1*, *Rere*, *Bach1*, *Siglec1*, *Txnip*, *Nrp1*, *Arhgap45*, *Inpp5d*, *Hs2st1*, *Slc8b1*, *Plxnb2*, *Man1c1*, *Atp13a2*, *Dusp7*, *Ptpra*, *Iffo2*, *Nxpe5*, *Ap1b1*, *Selenon*, *Slc43a2*, *Ssh2*, *Bmp2k*, *Tgfbr2*, *Gpr65*, *Pitpnc1*, *Pde7b*, *Abca9*, *Pon2*, *Agap1*, *Mctp1*, *Msl1*, *Stab1*, *Sntb2*, *Cnpy4*, *B3gnt3*, *Cd300ld*, *Zranb3*, *Gab1*, *Paqr3*

Cluster 6

Cln8, *Ccdc115*, *Mmp8*, *Gclm*, *Cluh*, *Ipo13*, *Arhgef10l*, *Blnk*, *Cx3cr1*, *Arhgap22*, *Pdgfb*

Cluster 7

Elmsan1, *Kdm3a*, *Pot1b*, *Fgd3*, *Fam210a*, *Klhl24*, *Zzz3*, *Tob2*, *Sh3tc1*, *Mbp*, *Slf2*, *Uprt*, *Mib1*, *Ogt*, *Zfp445*, *Samd8*, *Epm2aip1*, *Top2a*, *Nr2c2*, *Atrn*, *Dnmt3a*, *Zmiz1*, *Thbs1*, *Zfp292*, *Hook3*, *Fnip2*

Cluster 8

Rrad, *Gclc*, *Sephs2*, *Pygo2*, *Ptgfrn*, *Lrch4*, *Ubfd1*, *Ptgs1*, *Gdf3*, *Ciita*, *Lrrc8d*, *Fam76a*

Cluster 9

Tpm1, *Parvg*, *Dipk1a*, *6430548M08Rik*, *Serpinb6b*, *Nptx1*, *Rnf128*, *Gpcpd1*, *Rassf8*, *Scd2*, *B3galnt1*, *Slc17a5*, *Nlrp1b*, *Ccr2*, *Nlrp1c-ps*, *Prkci*, *Sema6d*, *Kcnk6*, *Cdkn1b*, *Pde4dip*, *Zadh2*, *Dipk2a*, *Dvl2*, *Cog6*, *Pisd-ps3*, *Cspg4*, *Mbd6*, *Arfgef3*, *Lactb2*, *F13a1*, *Camta2*, *Ptprs*, *Mapk7*, *P2ry1*, *Tbc1d2b*, *Trim59*, *Vps33a*, *Gcnt1*, *Hoxa1*, *Sorl1*, *Sort1*, *Mlec*, *Ube4b*, *Colec12*, *Abcb7*, *Gas2l3*, *Sh3pxd2a*, *Ppp1r9a*, *Dapp1*, *Slc36a4*, *Cfh*, *Rmnd5a*, *Tmem263*, *Adgre4*, *Pag1*, *Abi2*, *Apbb2*, *Pgap1*, *Kcnj2*, *Clcn3*, *Smc4*, *Spn*

A

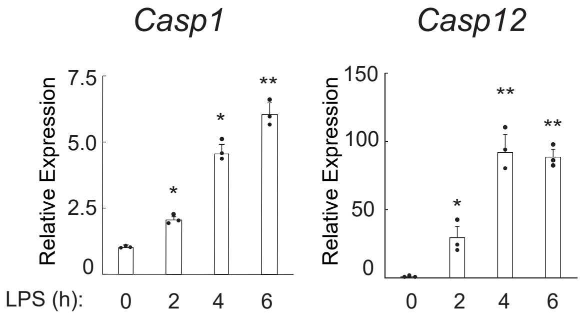

B

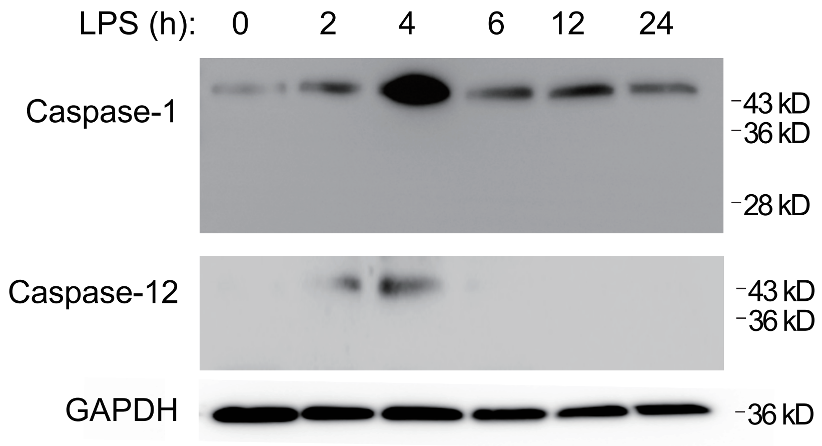

Suppl. Figure 1.

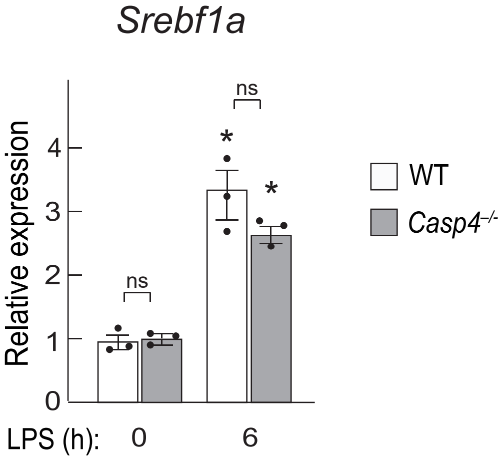

Suppl. Figure 2.

A

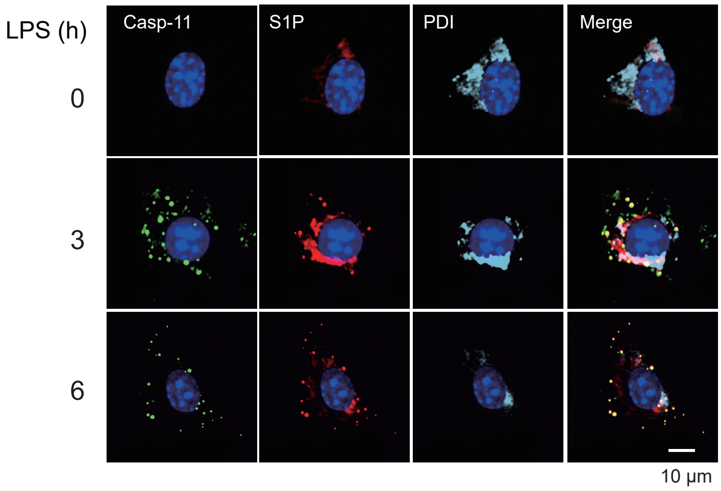

B

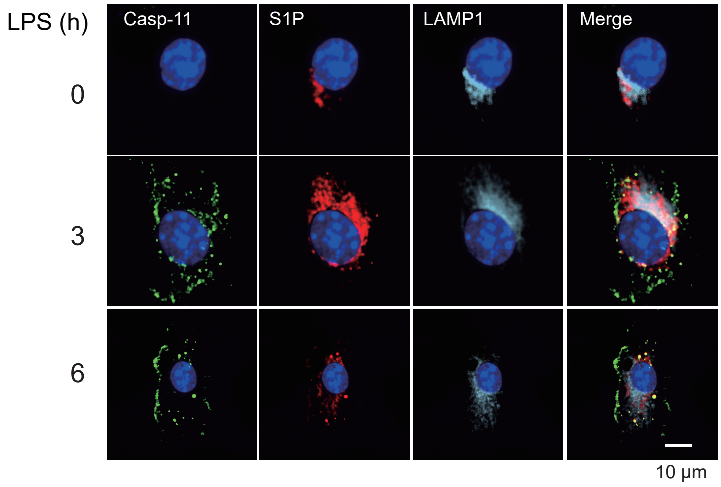

Suppl. Figure 3.

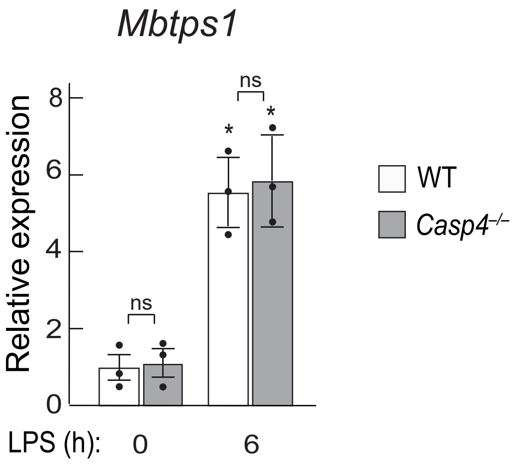

Suppl. Figure 4.

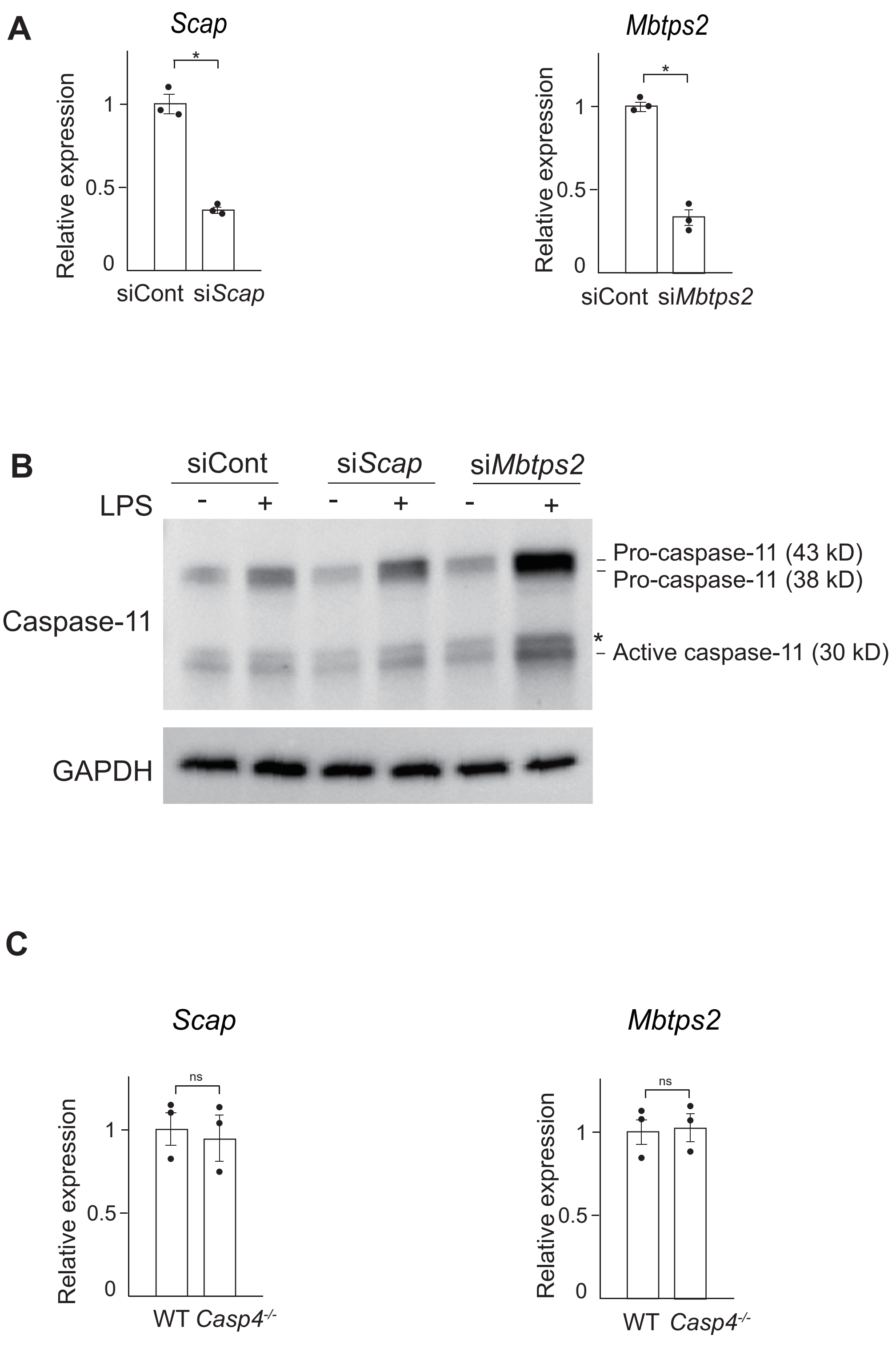

Suppl. Figure 5.

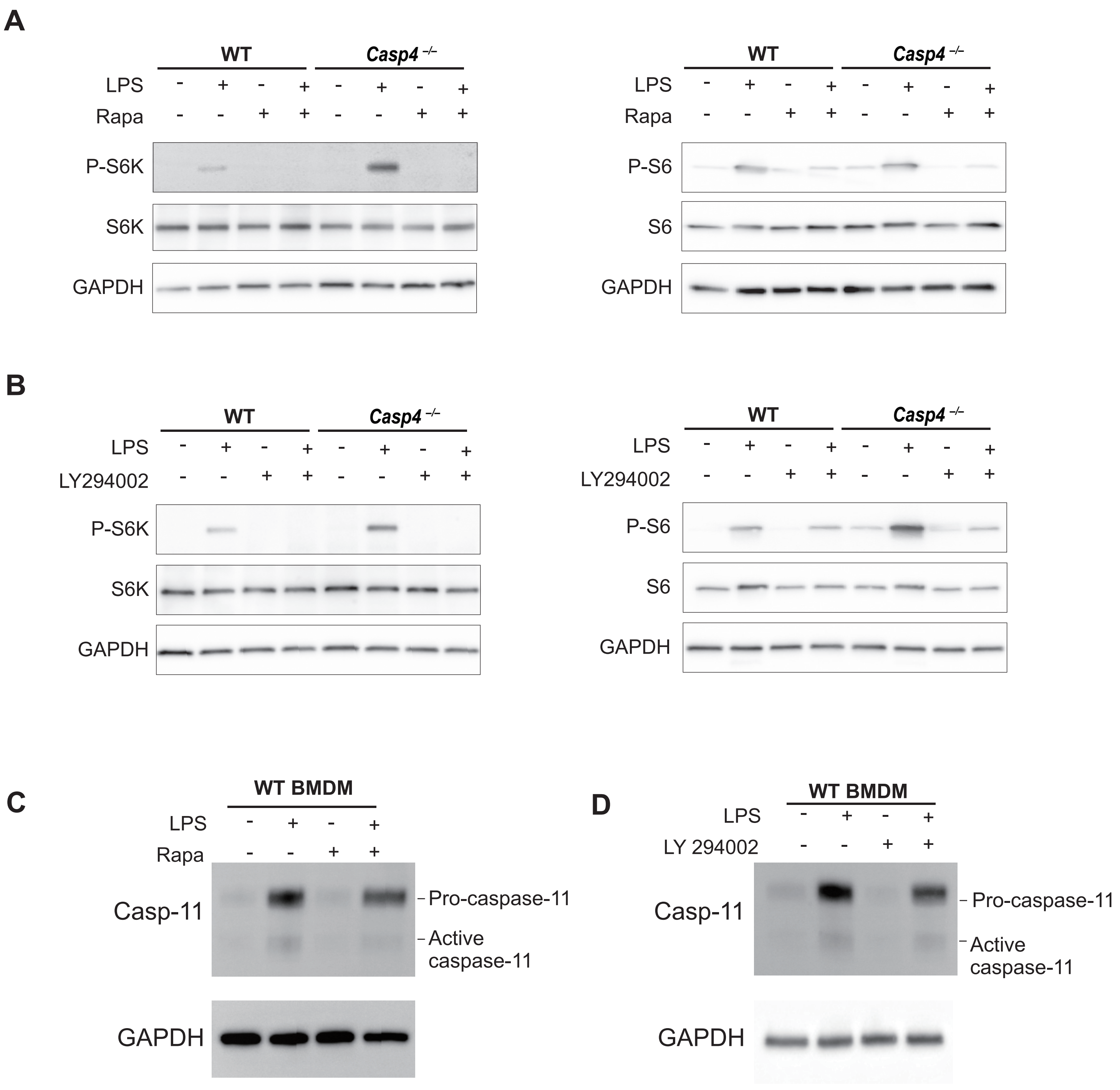

Suppl. Figure 6.

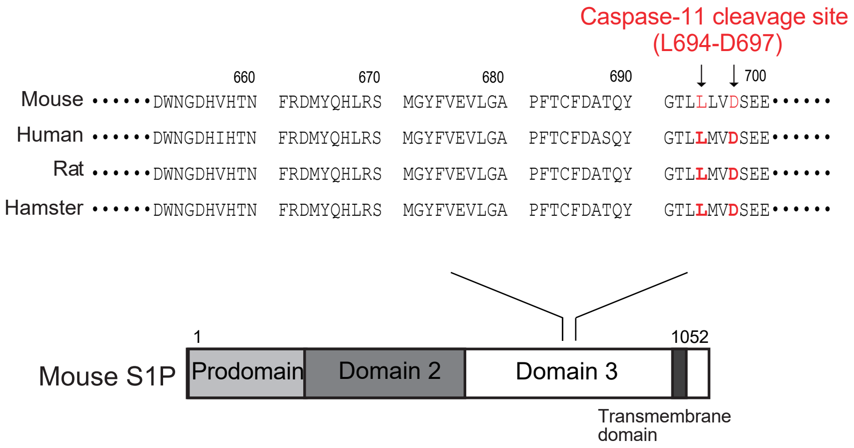

Suppl. Figure 7.

A

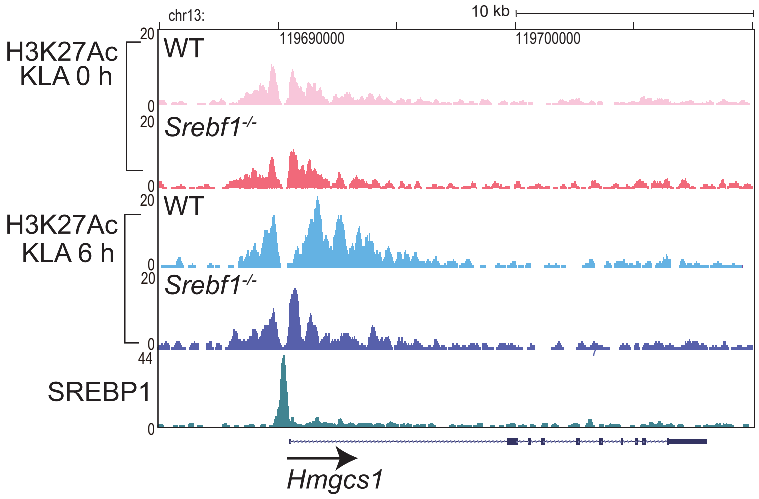

B

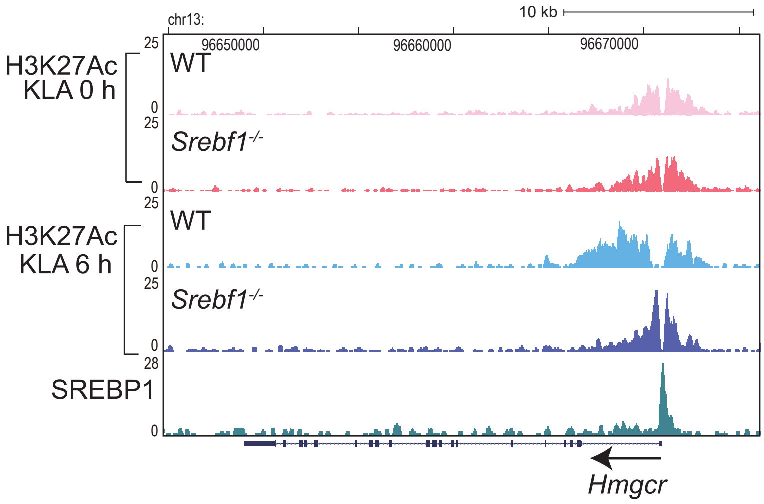

Suppl. Figure 8.

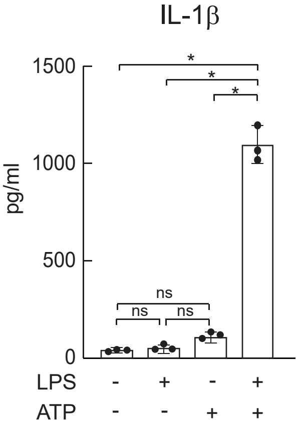

Suppl. Figure 9.
